# Multivariate sulfur-functionalized MOFs shaped into alginate spheres for robust and reusable multidye water remediation

**DOI:** 10.1039/d6dt01043a

**Published:** 2026-06-16

**Authors:** Jaume García, Paula Escamilla, Jesús Ferrando-Soria, Thais Grancha, Donatella Armentano, Emilio Pardo

**Affiliations:** a Instituto de Ciencia Molecular (ICMol), Universidad de Valencia 46980 Paterna Valencia Spain jesus.ferrando@uv.es emilio.pardo@uv.es; b Centro Singular de Investigación en Química Biolóxica e Materiais Moleculares (CiQUS), Universidade de Santiago de Compostela 15782 Santiago de Compostela Spain; c Dipartimento di Chimica e Tecnologie Chimiche (CTC), Università della Calabria Rende 87036 Cosenza Italy Donatella.armentano@unical.it

## Abstract

The removal of synthetic organic dyes from contaminated water remains a major environmental challenge, particularly under multicomponent and real-water conditions. Herein, we report a scalable adsorption platform based on sulfur-functionalized metal–organic frameworks (MOFs) for efficient multidye removal. Three isoreticular MOFs, including a multivariate analogue incorporating methylcysteine and methionine functionalities, were evaluated using real water (Turia River, Spain). The multivariate MOF shows faster adsorption kinetics and achieves quantitative dye removal, outperforming its monofunctional counterparts. For practical implementation, the MOFs were immobilized into calcium alginate spherical beads (≈50 wt% loading), yielding mechanically robust composites that preserve crystallinity and porosity. The shaped materials retain high adsorption efficiency and can be reused over multiple cycles without performance loss. Under continuous-flow solid-phase extraction conditions, the system achieves >99% removal for all dyes, enabling rapid and effective water decontamination.

## Abstract 2

A family of sulfur-functionalized MOFs enables the rapid, quantitative removal of multiple dyes from water. Shaped into calcium alginate beads, they achieve >99% dye removal under continuous-flow conditions, and exhibit excellent reusability.

## Graphical abstract

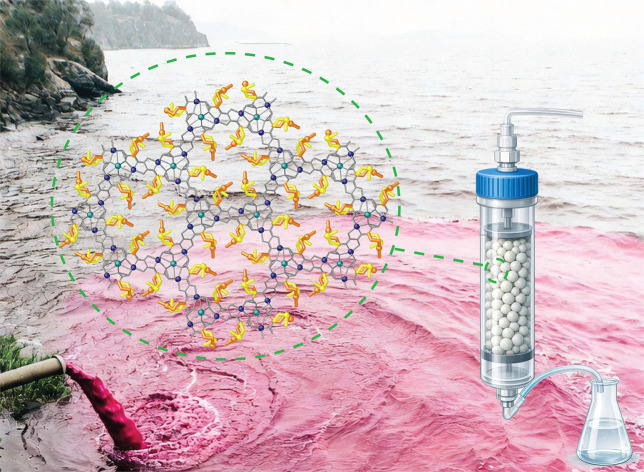

## 1. Introduction

The contamination of water resources by synthetic organic dyes^1^ represents a persistent environmental challenge due to their extensive use in textile, paper, pharmaceutical and bioimaging industries.^2^ Even at very low concentrations, dyes impart intense coloration to water streams, reducing light penetration and adversely affecting aquatic ecosystems,^3^ while many of them also present toxic or mutagenic effects.^4^ The chemical stability and structural diversity of dyes further complicate their removal by conventional treatment methods.^5^ In this context, the development of efficient, reusable and sustainable strategies for dye removal remains a priority for modern water remediation technologies.^6^

Among the different approaches explored for dye removal,^5,6^ adsorption^7,8^ stands out as an attractive and widely applicable strategy owing to its operational simplicity, high efficiency and compatibility with existing treatment infrastructures.^9,10^ Importantly, adsorption-based processes can be implemented under mild conditions and do not generate harmful transformation byproducts.^11^ However, the sustainability and practical relevance of adsorption technologies critically depend on the availability of adsorbent materials that combine high uptake capacity, fast kinetics, long-term reusability and straightforward recovery from aqueous media.^11^

Metal–organic frameworks (MOFs)^12–15^ have emerged as a versatile class of adsorbent materials for water remediation due to their exceptionally high surface areas, well-defined porosity and modular chemical tunability.^16–19^ The possibility of incorporating specific functional groups into MOF structures has enabled the rational design of materials with enhanced affinity toward targeted pollutants,^20,21^ including organic dyes^22,23^ and other emerging contaminants.^24^ Despite these advantages, most MOF-based adsorption studies are often conducted using microcrystalline powders,^25^ which pose significant challenges in terms of handling, separation and scalability.^25^ These limitations strongly hinder the translation of highly active MOFs from laboratory studies to practical water treatment systems.^26^

To address these challenges, considerable effort has been devoted to shaping^27–29^ MOFs into macroscopic and mechanically robust forms, such as mixed-matrix membranes,^30^ polymer foams^31^ or pelletized composites.^32^ These approaches represent important steps toward practical implementation, yet they often involve complex fabrication processes and may compromise the intrinsic properties of the MOF through pore blockage, reduced accessibility or slower adsorption kinetics.^27^ Consequently, there remains a clear need for simple, low-cost and scalable processing strategies that enable MOFs to be deployed in macroscopic forms without sacrificing their adsorption performance or structural integrity.^27–29^

Calcium alginate^33^ offers a particularly attractive platform for this purpose. As a naturally derived, inexpensive and environmentally benign biopolymer, alginate can be processed in aqueous media under mild conditions, aligning well with the principles of sustainable chemistry.^34^ Upon crosslinking with divalent cations, alginate readily forms mechanically stable spherical beads^35^ that can be easily handled, recovered and integrated into flow-through systems such as solid-phase extraction (SPE). These features make alginate-based spheres highly appealing for water remediation applications.^36^ Nevertheless, demonstrating that highly active MOFs can be incorporated into alginate matrices while fully retaining their crystallinity, porosity and adsorption efficiency remains a critical challenge.^36^

In parallel with processing considerations, the molecular-level design of MOFs plays a crucial role in achieving high adsorption performance. Multivariate MOFs^37,38^ (MTV-MOFs), which incorporate multiple functional groups within a single framework, have attracted growing attention due to their ability to create chemically diverse adsorption environments and promote synergistic interactions with target pollutants.^39^ Such functional heterogeneity is particularly advantageous for the removal of complex contaminant mixtures, where different adsorption mechanisms may operate simultaneously. However, the integration of MTV-MOFs into processable and sustainable adsorbent platforms has so far been scarcely explored.^40^

**Fig. 1:**
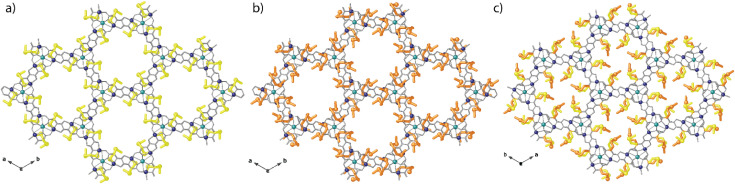
Perspective views of the crystal structures of MOFs 1 (a), 2 (b) and MTV-MOF 3 (c). Metal atoms are represented as blue (Ca^II^) and cyan (Zn^II^) spheres whereas organic ligands are represented as thin grey sticks with the exception of amino acid residues, which are depicted as yellow (–CH_2_SCH_3_ from *S*-methyl-l-cysteine) and orange (–CH_2_CH_2_SCH_3_ from l-methionine) thick sticks to emphasize the functionalization of the channels.

Herein, we report a systematic study combining molecular design and sustainable processing to advance the practical use of MOFs in water remediation. For this purpose, three previously reported^40–43^ sulfur-functionalized MOFs –including two monofunctional materials bearing methylcysteine or methionine residues and a multivariate MOF combining both functionalities within the same framework (Scheme S1 and Fig. 1)– have been selected. Their adsorption performance is evaluated toward a multidye complex water matrix, revealing a clear enhancement for the MTV-MOF that is attributed to functional synergy, in line with previous observations.^39,44^ Crucially, these MOFs are processed into calcium alginate spheres that preserve the crystallinity, porosity and adsorption efficiency of the corresponding powdered materials. The resulting spherical adsorbents exhibit fast and quantitative dye removal,^45,46^ excellent recyclability and structural robustness, highlighting alginate-based shaping as a sustainable and effective strategy to bridge advanced MOF design with realistic water remediation technologies.^16^

## 2. Results and discussion

In this work, we investigate the adsorption performance and processability of sulfur-functionalized metal–organic frameworks (MOFs) for water remediation applications. Thus, three previously reported oxamidato-based MOFs^40,42^ derived from the amino acids *S*-methyl-l-cysteine and l-methionine –including two monofunctional materials bearing exclusively *S*-methyl-l-cysteine (1) or l-methionine (2) residues and also a multivariate MOF (3) combining 50% of both functionalities (–CH_2_SCH_3_ and –CH_2_CH_2_SCH_3_) in a 1 : 1 ratio within the same framework (Fig. 1)– were employed. The three materials were selected to enable a systematic comparison between monofunctional and multivariate pore environments and to assess the impact of functional heterogeneity on dye adsorption performance.

The study was conducted with three main objectives: (i) to evaluate the efficiency of the powdered crystalline MOFs toward the capture of organic dyes from aqueous media, with particular emphasis on the role of dual functionalization in the multivariate MOF 3; (ii) to process these MOFs into calcium alginate spheres in order to obtain macroscopic, mechanically robust composites suitable for practical handling and implementation in capture devices; and (iii) to assess whether the adsorption properties observed for the powdered materials are fully retained upon shaping, thereby demonstrating the effective translation of intrinsic MOF performance into processed and reusable adsorbents.

### 2.1. Synthesis and characterization

MOFs 1 and 2, together with the multivariate analogue 3, were prepared following the same previously established synthetic procedure that enables their isolation as gram-scale polycrystalline powders as well as single crystals suitable for structural analysis using slow diffusion methods (see Experimental section). The materials correspond to the formulations {Ca^II^Zn^II^_6_[(*S*,*S*)-mecysmox]_3_(OH)_2_(H_2_O)}·12H_2_O^47^ (1), {Ca^II^Zn^II^_6_[(*S*,*S*)-methox]_3_(OH)_2_(H_2_O)}·16H_2_O^42^ (2) and {Ca^II^Zn^II^_6_[(*S*,*S*)-methox]_1.5_[(*S*,*S*)-mecysmox]_1_._5_(OH)_2_(H_2_O)}·12H_2_O^40^ (3), where Mecysmox and methox denote bis[(*S*)-methylcysteine]oxalyldiamide and bis[(*S*)-methionine]oxalyldiamide ligands, respectively.

Prior to evaluating their adsorption performance, the crystallinity, phase purity and permanent porosity of 1–3 were assessed using C, H, S, N analyses (see Experimental section), powder X-ray diffraction (PXRD) and N_2_ adsorption measurements on freshly prepared polycrystalline samples. The PXRD patterns (Fig. S1 and S2, see SI) confirm the phase purity of all materials and their isoreticular relationship. Nitrogen adsorption isotherms recorded at 77 K closely match those previously reported for 1–3 ^40,42^ (Fig. S3), indicating preservation of the porous frameworks. The Brunauer–Emmett–Teller (BET) surface areas^48^ were determined to be 725, 451 and 664 m^2^ g^−1^ for MOFs 1–3, respectively.

Fig. 1 depicts the crystal structures of MOFs 1–3, which are all isomorphous and crystallize in the chiral *P*6_3_ space group of the hexagonal system. The three materials are based on chiral three-dimensional bimetallic (Ca^II^–Zn^II^) networks featuring one-dimensional medium-sized hexagonal channels, whose internal surfaces are functionalized by the corresponding sulfur-containing amino acid residues.

In particular, the crystal structure of the multivariate framework 3 (Fig. 1c) clearly reveals medium-sized hexagonal channels simultaneously decorated by methionine and methylcysteine residues, unambiguously confirming the incorporation of both ligands within the same framework and giving rise to a chemically heterogeneous pore environment. As in the parent monofunctional materials 1 and 2, the framework of 3 is structurally robust, with conformational flexibility largely confined to the pendant thioether side chains lining the pores. Based on previous studies on related sulfur-functionalized MOFs, such flexible thioether functionalities present in 1–3 are expected to engage in a range of noncovalent interactions with organic guests, including van der Waals contacts, hydrogen bonding, and σ-hole interactions. These interactions are anticipated to provide adaptable adsorption sites while preserving the structural integrity of the framework.^49–52^

### 2.2. Capture experiments of powdered samples

At this stage, the capture efficiencies of polycrystalline powdered samples of 1–3 were evaluated using a multidye environmental aqueous solution (Turia river, Valencia, Spain, 39.5463° N, −0.5477° W) containing methylene blue (MB), brilliant green (BG), pyronine Y (PY) and auramine O (AO) (10 mg L^−1^ each, Scheme S2). The pH of the resulting multidye solution was measured to be 7.4. Dye removal was monitored by UV–vis spectroscopy as a function of time (1–120 min), with concentrations quantitatively determined using calibration curves obtained from single-component aqueous solutions at the characteristic absorption maxima of each dye (Fig. S4). To ensure accurate quantification within the linear Beer–Lambert regime, aliquots (1 mL) withdrawn at selected time intervals were diluted to 3 mL prior to analysis, resulting in absorbance values below 1.0; measured concentrations were subsequently corrected for the dilution factor and used to calculate dye removal efficiencies relative to the initial concentration.

**Fig. 2:**
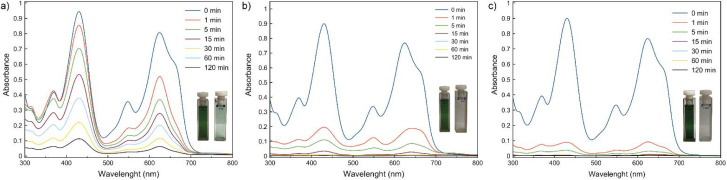
Evolution with time of the UV-Vis absorption spectra of a multidye solution containing 10 ppm solutions of auramine O, brilliant green, methylene blue and pyronin Y in real water samples from Turia river in the presence of 50 mg of MOF **1** (a), MOF **2** (b), MTV-MOF **3** (c). The photographs show the colours of the solutions at the beginning (left) and after 30 minutes of exposure of the multidye solution with the corresponding (MTV)-MOF (right).

Under identical experimental conditions, MOF 1 showed only a moderate dye uptake (Fig. 2a and S5 and Table S1). In turn, MOF 2 –bearing larger and more flexible –CH_2_CH_2_SCH_3_ side chains– exhibited significantly faster kinetics and higher removal efficiencies, being capable to remove virtually all four dyes in only 30 min (Fig. 2b and S6 and Table S1). This behaviour is in line with previous observations reported for the analogous Cu_6_Ca-based MOF.^46^ Remarkably, the multivariate MOF 3 displayed an even better performance, achieving quantitative removal of all dyes within only 15 min (Fig. 2c and S7 and Table S1). Although the precise molecular origin of this behaviour cannot be unequivocally established from the present data, the coexistence of distinct sulfur-functionalized environments within MTV-MOF 3 may contribute to the observed improvement in adsorption kinetics and dye removal efficiency, as observed previously.^39^

To further evaluate whether the improved performance of MTV-MOF 3 arises simply from the simultaneous presence of both monofunctional components, additional control experiments were performed using a physical 1 : 1 mixture of MOFs 1 and 2 under identical conditions (Fig. S8 and Table S2). The adsorption behaviour of the physical mixture was found to be approximately intermediate between those of the parent materials and significantly inferior to that of MTV-MOF 3. These results indicate that the enhanced adsorption performance of MTV-MOF 3 cannot be reproduced by a simple physical combination of MOFs 1 and 2 and are consistent with a beneficial effect associated with the chemically heterogeneous environment generated within the multivariate framework.

### 2.3. Preparation of calcium alginate-based spheres containing MOFs 1–3

**Fig. 3:**
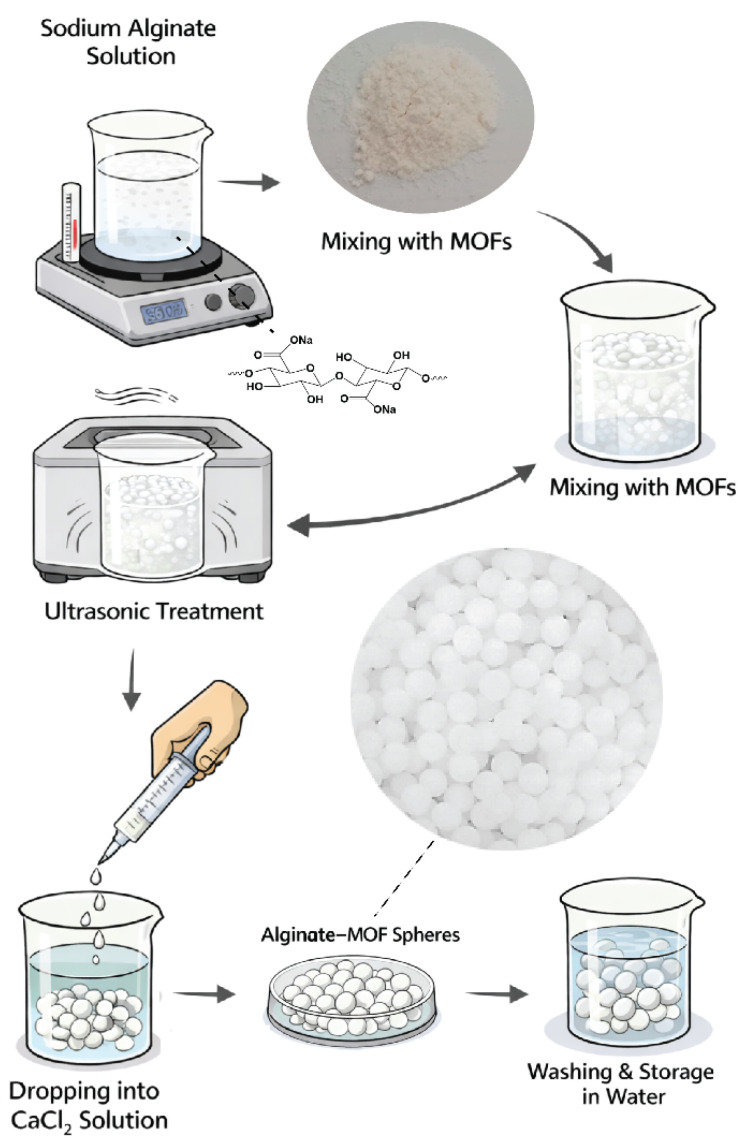
Synthetic route for the preparation of calcium alginated-based spheres containing MOFs 1–3 (named CAS-1, CAS-2 and CAS-3, respectively).

Having established the intrinsic adsorption performance of the powdered MOFs toward the multidye system, we next addressed their processing into macroscopic and mechanically robust forms suitable for practical deployment.^27–29^ To improve material handling, facilitate recovery from aqueous media and enable potential integration into capture devices, MOFs 1–3 were incorporated into calcium alginate matrices to form spherical composite beads (Fig. 3).^35^ The resulting hybrid calcium alginate spheres are hereafter referred to as CAS-1, CAS-2 and CAS-3, respectively. Alginate-based shaping has been previously explored as a means to enhance the applicability of MOF adsorbents, with reported examples including *in situ* ZIF-8/alginate^53^ (and other MOFs^54–56^) gels for pharmaceutical removal,^53^ MOF–alginate beads for ion capture from seawater,^57^ and UiO-66 or magnetic MOF composites for heavy metal and dye adsorption.^58–61^ These studies highlight the promise of alginate as a sustainable processing platform for MOF-based adsorbents.

Nevertheless, many reported systems rely on MOFs with limited chemical tuneability, target single-component pollutants, or exhibit partial loss of porosity and adsorption efficiency upon encapsulation within the polymer matrix.^62^ Moreover, adsorption studies are most often conducted under dispersive batch conditions, while demonstrations under continuous-flow or solid-phase extraction (SPE) operation^50–52^ –more relevant for realistic and time-efficient water treatment– remain scarce. In particular, systematic evidence showing that shaping strategies can fully preserve the intrinsic performance of highly crystalline and functionally complex MOFs, including under multicontaminant adsorption conditions, repeated use and flow-through operation, is still limited. In this context, alginate spheres provide an ideal platform to assess whether advanced MOF designs, including multivariate frameworks, can be translated into processable, robust and reusable adsorbents without compromising adsorption efficiency or structural integrity.

In this context, we focused our efforts on the preparation of alginate-based hybrid spheres incorporating the highest possible loading of MOFs 1–3, while preserving the adsorption properties observed for the corresponding polycrystalline powders (see Experimental section and Fig. 3). A sodium alginate (SA) aqueous solution was prepared by dissolving 0.25 g of SA in 15 mL of deionized water under stirring at 60 °C for 1 h in a thermostated water bath. Subsequently, 0.25 g of MOFs 1, 2 and 3, respectively, were added to the alginate solutions, in separate experiments, under continuous stirring, affording homogeneous white gelatinous suspensions. To eliminate entrapped air bubbles and ensure uniform dispersion of the MOF particles, the suspensions were subjected to ultrasonic water-bath treatment for 30 min. The resulting mixtures were then added dropwise, using a 25 mL syringe, into a 4 wt% aqueous CaCl_2_ solution, leading to instantaneous crosslinking and the formation of spherical hydrogel beads. Finally, the obtained alginate spheres were thoroughly washed with deionized water for 24 h and stored in water (Fig. S9) prior to further characterization and adsorption studies.

The morphology, composition and structural integrity of the alginate-based hybrid spheres CAS-1, CAS-2 and CAS-3 were thoroughly characterized by a combination of powder X-ray diffraction (PXRD), nitrogen adsorption measurements, scanning electron microscopy (SEM), energy-dispersive X-ray spectroscopy (SEM–EDX) and elemental (CHNS) analyses. PXRD patterns recorded for the freshly prepared spheres match well with those of the corresponding powdered MOFs (Fig. S10), confirming that the crystalline frameworks are preserved during the alginate encapsulation process. Importantly, PXRD measurements performed on the spheres after storage in water for three months reveal no detectable structural degradation, highlighting the excellent hydrolytic stability of the composites under prolonged aqueous conditions (Fig. S11).

**Fig. 4:**
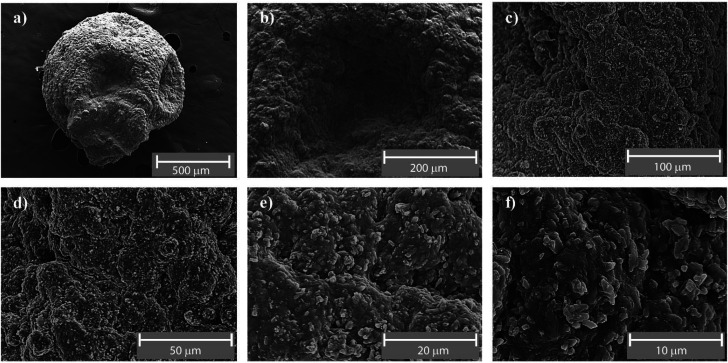
(a) SEM image of a single calcium alginate sphere of CAS-1 (a) and (b–f) different zoomed areas of the sphere.

Nitrogen adsorption isotherms measured at 77 K show that the alginate spheres retain permanent porosity, with maximum N_2_ uptake values corresponding to approximately 50% of those observed for the powdered materials (Fig. S12). This result is fully consistent with the MOF loading in the spheres, which was determined to be *ca.* 50 wt% by both CHNS and SEM–EDX analyses (Fig. S13–S15) –which show a very homogeneous distribution of MOFs’ particles within the spheres– and indicates that the accessible porosity of the MOFs is largely preserved upon shaping. SEM images further reveal the formation of uniform spherical beads with well-defined morphology, in which the MOF microcrystals are homogeneously embedded within the alginate matrix and remain clearly discernible (Fig. 4 and S16, S17). Collectively, these results demonstrate that alginate-based processing enables high MOF loadings while preserving crystallinity, porosity and long-term stability, thereby providing robust and processable adsorbents suitable for water remediation applications.

### 2.4. Capture experiments of calcium alginate spheres

**Fig. 5:**
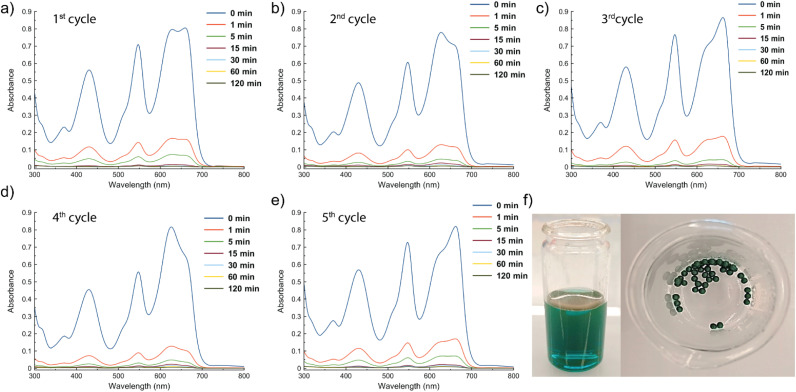
Time evolution of the UV–Vis absorption spectra of a multidye solution containing 10 mg L^−1^ each of auramine O, brilliant green, methylene blue, and pyronin Y in real water samples from the Turia river, in the presence of 50 mg of CAS-3, over five consecutive cycles (a–e). (f) Photograph of the multidye solution before the capture experiments (left) and image of CAS-3 spheres together with the resulting colourless solution after 120 minutes in the fifth capture cycle (right).

The adsorption performance of the alginate-based spheres containing MOFs (CAS-1, CAS-2 and CAS-3) was next evaluated under batch conditions using the same multidye aqueous solution (AO, PY, BG and MB; 10 mg L^−1^ each) employed for the powdered materials. In these experiments, 50 mg of spheres were contacted –under continuous stirring– with 25 mL of the multidye solution, and the dye removal was monitored by UV–vis spectroscopy as a function of time. Notably, the alginate spheres exhibit adsorption profiles and overall removal efficiencies that closely reproduce (Fig. 5 and S18–S34 and Tables S3–S5) those observed for the corresponding powdered MOFs 1–3 (Fig. 2 and S5–S7), achieving quantitative dye removal within comparable timescales. A slightly slower uptake kinetics is observed for the spheres, which can be rationalized by their effective MOF content (*ca*. 50 wt%, corresponding to ∼25 mg of MOF per experiment), compared to the 50 mg of pure MOF used in the powder-based studies.

To evaluate the contribution of the alginate matrix itself, control experiments were performed using pristine calcium alginate spheres prepared under identical conditions but in the absence of MOFs. These control spheres exhibited only negligible dye uptake under the investigated conditions (Fig. S35 and Table S6), confirming that the adsorption performance of CAS-1, CAS-2 and CAS-3 is predominantly governed by the embedded MOF particles, while the alginate matrix mainly acts as a mechanically robust shaping and supporting medium.

Importantly, the alginate spheres display excellent reusability. Each material was subjected to five consecutive adsorption cycles, showing essentially unchanged capture efficiencies relative to the first cycle (Fig. 5 and S18–S34 and Tables S3–S5). Regeneration between cycles was achieved by simply suspending the spheres in a water/methanol (1 : 1 v/v) mixture for 30 s, followed by direct reuse without any additional treatment. To further evaluate the efficiency of the regeneration procedure, the washing solutions obtained after treatment of dye-loaded CAS materials with a water/methanol (1 : 1 v/v) mixture were analysed by UV–vis spectroscopy. Desorption efficiencies in the 45–60% range were observed for the different dyes after a contact time shorter than 30 s (Table S7), demonstrating the rapid release of a significant fraction of the adsorbed guests during the regeneration step. The preservation of adsorption performance over multiple cycles highlights the robustness of the alginate–MOF composites and demonstrates that shaping into spherical beads does not compromise the intrinsic adsorption properties of the parent MOFs. Moreover, the structural integrity of the best performing material embedded in alginate spheres (CAS-3), after the five consecutive capture cycles, was established by measuring PXRD patterns after the fifth cycle (Fig. S36).

The total adsorption capacities under the experimental conditions (*q*_e_) employed in this work were approximately 18 mg g^−1^ for CAS-1 and 20 mg g^−1^ for both CAS-2 and CAS-3 when referred to the total mass of the composite material. Considering the *ca.* 50 wt% MOF loading of the spheres, these values correspond to approximately 36 mg g^−1^ (CAS-1) and 40 mg g^−1^ (CAS-2 and CAS-3) when normalized to the actual MOF content. These values represent adsorption capacities under the experimental conditions employed and should not be interpreted as maximum adsorption capacities (*q*_max_) derived from adsorption isotherms.

Since CAS-1, CAS-2 and CAS-3 are hydrogel-based composites, they are intended to be stored and used in the hydrated state, whereas drying leads to irreversible shrinkage and hardening of the beads. Under hydrated conditions, the CAS materials retained their spherical morphology without visible swelling, deformation or disintegration after storage in water for more than three months (Fig. S37). This observation, together with the preservation of crystallinity after repeated adsorption/desorption cycles (Fig. S36), further demonstrates the excellent operational stability of the alginate–MOF composites.

To evaluate the influence of pH on the performance and stability of the best-performing material (CAS-3), additional adsorption experiments were conducted at pH 4 and pH 10. In both cases, the material retained high dye removal efficiencies (Fig. S38 and Table S8) and preserved its crystallinity after the adsorption experiments, as confirmed by PXRD analysis (Fig. S39). These results indicate that CAS-3 remains structurally stable and functional under moderately acidic and basic conditions.

**Fig. 6:**
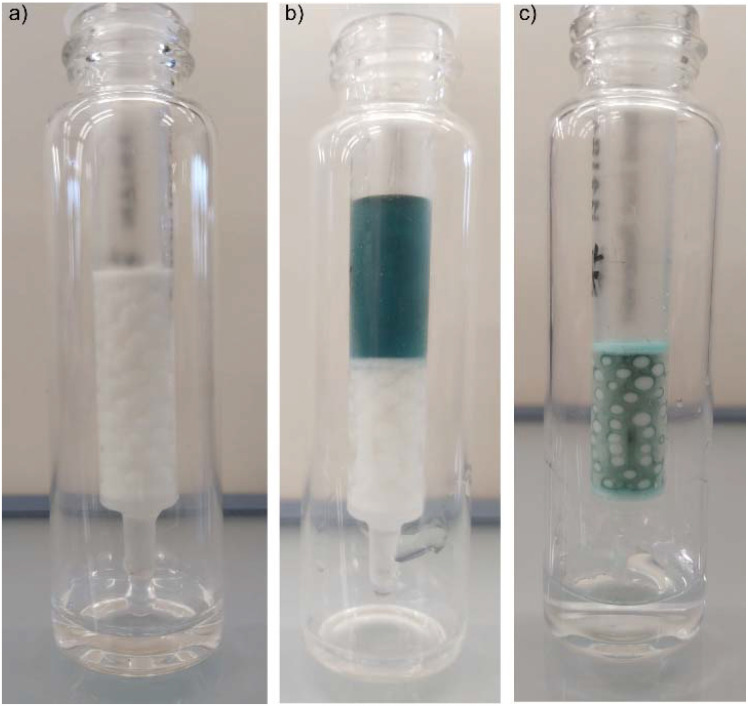
Photographs of the solid-phase extraction (SPE) setup showing the packed CAS-3 (a), the multidye aqueous solution prior to percolation (b), and the colorless solution collected after passing through the column (c).

Finally, the practical applicability of the alginate-based spheres was evaluated under continuous-flow conditions using a solid-phase extraction (SPE) setup (Fig. 6), where 25 mg of the best performing spheres (CAS-3) were packed into a 10 mL polypropylene column (Fig. 6a) and confined between two porous frits (0.5 cm pore size).

The resulting SPE device was then challenged with a 5 mL multidye aqueous solution (MB, BG, PY, and AO; 10 mg L^−1^ each) under gravity-flow conditions (Fig. 6b). The percolated solution exited the column colourless and was subsequently analysed by UV–vis spectroscopy. Remarkably, dye removal efficiencies exceeding 99% were obtained for all four dyes (Fig. S40 and Table S9), demonstrating quantitative decontamination under flow-through conditions.

Compared to batch adsorption experiments, the SPE configuration enables significantly faster processing while maintaining –and even slightly improving– removal efficiencies. This enhanced performance can be attributed to the establishment of multiple dynamic adsorption equilibria along the packed bed, which promote efficient contact between the solution and the active MOF sites. The effectiveness of this process is visually illustrated in Fig. S6, which shows the packed CA spheres (Fig. S6a), the deep green contaminated solution prior to entering the column (Fig. S6b), and the completely colourless solution exiting the column (Fig. S6c). Overall, these results highlight the suitability of alginate-shaped multivariate MOFs for continuous and time-efficient water remediation, further underscoring the advantages of combining advanced MOF design with sustainable shaping strategies.

In order to place the performance of the present system into context, a comparison with representative MOF–alginate composites previously reported for pollutant removal is provided in Table 1. Although direct quantitative comparisons are inherently complicated by differences in pollutant identity, concentration, adsorbent loading and operating conditions, the comparison reveals that the present CAS materials combine several features that are rarely reported simultaneously, including rapid multidye removal, operation in real river water, excellent recyclability and successful implementation under continuous-flow SPE conditions.

**Table 1:** Representative MOF–alginate composites reported for pollutant removal and comparison with the present work

Material	MOF	Pollutant system	Water matrix	Reusability	Flow operation	Key observation	Ref.
ZIF-8/alginate beads	ZIF-8	Pharmaceutical pollutant	DI water	Yes	No	*In situ* gel formation; pollutant removal	53
MOF/alginate beads	Various MOFs	Single-component pollutants	DI water	Yes	No	General proof-of-concept for MOF immobilization	54–56
MOF–alginate beads	MOF composite	Metal ions from seawater	Seawater	Yes	No	Ion capture from complex matrix	57
UiO-66/alginate beads	UiO-66	Heavy metals/dyes	DI water	Yes	No	Retained adsorption after shaping	58
Magnetic MOF/alginate composites	Magnetic MOF	Dyes	DI water	Yes	No	Easy magnetic recovery	59–61
CAS-3 (this work)	MTV-MOF	Multidye system (AO, PY, BG, MB)	Real river water (Turia)	5 cycles	Yes (SPE)	Quantitative removal, fast kinetics, flow-through operation	This work

## 3. Conclusions

This work demonstrates how the combination of rational MOF design and sustainable shaping strategies can enhance the practical applicability of MOF-based adsorbents for real-world water remediation. Two monofunctional sulfur-functionalized MOFs (1 and 2) and a multivariate analogue (3) were investigated toward the removal of a multidye aqueous system. The MTV-MOF 3 displays slightly faster adsorption kinetics and superior capture performance, underscoring the potential benefits of chemically heterogeneous pore environments for multicomponent pollutant removal.

Although the precise origin of this performance enhancement cannot be unequivocally established, it is likely associated with the coexistence of linkers of different length and flexibility within the framework. The elongated methionine-based ligand present in MOF 2 may contribute enhanced conformational adaptability, potentially facilitating kinetically favoured sequestration of contaminants. In parallel, the shorter methylcysteine-derived linker (MOF 1), which preserves similar chemical functionality, may provide increased accessible volume, enabling dye molecules to adopt more favourable orientations within the channels. The combination of these features in the MTV-MOF may therefore promote cooperative host–guest interactions arising from both flexible anchoring sites and accessible pore space. Such a heterogeneous environment is expected to favour the stabilization of guest species through a network of non-covalent interactions, including van der Waals contacts and σ-hole interactions, consistent with the rapid and quantitative removal observed experimentally.

Importantly, all MOFs were successfully processed into calcium alginate spheres with high MOF loadings (*ca.* 50%), while fully retaining crystallinity, permanent porosity and hydrolytic stability. The alginate-shaped materials reproduce the adsorption performance of the powdered MOFs, showing fast and quantitative dye removal under batch conditions, excellent reusability over multiple cycles and preserved structural integrity. Furthermore, solid-phase extraction experiments confirm quantitative dye removal under continuous-flow operation, enabling faster and more efficient decontamination compared to dispersive adsorption.

Overall, this study highlights alginate-based shaping as a simple, sustainable and effective route to translate advanced and functionally complex MOFs into robust, reusable and flow-compatible adsorbents, bridging the gap between molecularly designed MOFs and realistic water remediation technologies.

## Conflicts of interest

There are not conflicts of interest to declare.

## Supplementary Material

DT-055-D6DT01043A-s001

## Data Availability

The data supporting this article have been included as part of the supplementary information (SI). Any other data would be available upon reasonable request from the authors. Supplementary information is available. Preparation and physical characterization data. Additional capture experiment details. Additional Figs. S1–40, Schemes S1 and S2 and Tables S1–S9. See DOI: https://doi.org/10.1039/d6dt01043a.
