# Supplementary material for: Multivariate sulfur-functionalized MOFs shaped into alginate spheres for robust and reusable multidye water remediation
Source: Dalton Trans. 2026 Jun 16;55(26):9949–57. doi: 10.1039/d6dt01043a (PMC13280889; doi:10.1039/d6dt01043a)
Supplement: DT-055-D6DT01043A-s001 [file DT-055-D6DT01043A-s001.pdf]

Supplementary Information (SI) for Dalton Transactions.
This journal is © The Royal Society of Chemistry 2026

Electronic Supporting Information (ESI):

Multivariate Sulfur-Functionalized MOFs Shaped into Alginate Spheres for Robust and Reusable Multidye Water Remediation

Jaume García,[‡a] *Paula Escamilla,*[‡b] *Jesús Ferrando-Soria,*[*a] *Thais Grancha,*[a] *Donatella Armentano*[*c] *and Emilio Pardo*[*a]

[a] Instituto de Ciencia Molecular (ICMol), Universidad de Valencia, 46980 Paterna, Valencia, Spain.

[b] Centro Singular de Investigación en Química Biolóxica e Materiais Moleculares (CiQUS), Universidade de Santiago de Compostela, 15782 Santiago de Compostela, Spain.

[c] Dipartimento di Chimica e Tecnologie Chimiche (CTC), Università della Calabria, Rende 87036, Cosenza, Italy.

Experimental Section

Physical Techniques. Elemental (C, H, S, N) analyses were performed at the Microanalytical Service of the Universitat de València. FT–IR spectra were recorded on a Perkin-Elmer 882 spectrophotometer as KBr pellets.

Materials. All chemicals were of reagent grade quality. They were purchased from commercial sources and used as received. MOFs $\{Ca^{II}Zn^{II}_6[(S,S)$-Mecysmox$]_3(OH)_2(H_2O)\} \cdot 12H_2O$[1] (**1**), $\{Ca^{II}Zn^{II}_6[(S,S)$-methox$]_3(OH)_2(H_2O)\} \cdot 16H_2O$[2] (**2**) and $\{Ca^{II}Zn^{II}_6[(S,S)$-methox$]_{1.5}[(S,S)$-Mecysmox$]_{1.5}(OH)_2(H_2O)\} \cdot 12H_2O$[3] (**3**) were prepared, as previously reported, by following the same synthetic procedure:

Preparation of $\{Ca^{II}Zn^{II}_6[(S,S)$-Mecysmox$]_3(OH)_2(H_2O)\} \cdot 12H_2O$ (1), $\{Ca^{II}Zn^{II}_6[(S,S)$-methox$]_3(OH)_2(H_2O)\} \cdot 16H_2O$ (2) and $\{Ca^{II}Zn^{II}_6[(S,S)$-methox$]_{1.5}[(S,S)$-Mecysmox$]_{1.5}(OH)_2(H_2O)\} \cdot 12H_2O$ (3): The corresponding organic proligand: H_2Me_2-(*S*,*S*)-Mecysmox (4.22 g, 12.0 mmol), H_2Me_2-(*S*,*S*)-methox (4.53 g, 12.0 mmol) or an equimolar mixture of both ligands (6.0 mmol of each), for the obtention of MOFs **1**, **2** and **3**, respectively, was suspended in 40 mL of water and treated with a 25% methanolic solution of Me_4NOH (14.5 mL, 50.0 mmol) until complete dissolution. Then, another aqueous solution (40 mL) containing $CaCl_2$ (4.0 mmol) and $ZnCl_2$ (24.0 mmol) was added dropwise, under stirring, to the corresponding ligand solution. After further stirring for 10 h, at room temperature, white polycrystalline powders were obtained and collected *via* filtration and dried with methanol. **MOF 1**: Yield: 5.92 g, 89%; Anal.: calcd. for $C_{30}H_{64}N_6O_{33}S_6Zn_6Ca$ (1661.7): C, 21.68; H, 3.88; N, 5.06; S, 11.58%. Found: C, 21.67; H, 3.72; N, 5.06; S, 11.47%. IR (KBr): ν = 1613 and 1608 cm^{-1} (C=O). **MOF 2**: Yield: 5.74 g, 79%; Anal.: calcd for $C_{36}Zn_6CaH_{84}S_6N_6O_{37}$ (1817.88): C, 23.79; H, 4.66; N, 4.62; S, 10.58%. Found: C, 24.01; H, 4.51; N, 4.63; S, 10.51%. IR (KBr): ν = 1611 cm^{-1} (C=O). **MTV-MOF 3**: Yield: 5.52 g, 81%; Anal.: calcd for $C_{33}Zn_6CaS_6H_{70}N_6O_{33}$ (1703.74): C, 23.26; H, 4.14; S, 11.29; N, 4.93%. Found: C, 23.31; H, 4.13; S, 11.31; N, 4.99%. IR (KBr): ν = 1607 cm^{-1} (C=O).

Preparation of calcium alginate (CA) spheres of MOFs 1-3 (CAS-1, CAS-2 and CAS-3). A sodium alginate (SA) aqueous solution was prepared by dissolving 0.25 g of SA in 15 mL of deionized water under stirring at 60 °C for 1 h in a thermostated water bath. Subsequently, 0.25 g of MOFs **1**, **2** and **3**, respectively, were added to the alginate

solutions, in separate experiments, under continuous stirring, affording homogeneous white gelatinous suspensions. To eliminate entrapped air bubbles and ensure uniform dispersion of the MOF particles, the suspensions were subjected to ultrasonic water-bath treatment for 30 min. The resulting mixtures were then added dropwise, using a 25 mL syringe, into a 4 wt% aqueous $CaCl_2$ solution, leading to instantaneous crosslinking and the formation of spherical hydrogel beads. Finally, the obtained alginate spheres were thoroughly washed with deionized water for 24 h and stored in water. **CAS-1:** Yield: 0.48 g, 94%; Anal.: calcd. for $C_{81}H_{123.5}N_6O_{84}S_6Zn_6Ca_{5.25}$ (3320.5): C, 29.31; H, 3.75; N, 2.53; S, 5.79%. Found: C, 28.67; H, 3.72; N, 2.67; S, 5.61%. **CAS-2**: Yield: 0.45 g, 91%; Anal.: calcd for $C_{90}Zn_6Ca_{5.5}H_{147}S_6N_6O_{91}$ (3574.3): C, 30.24; H, 4.14; N, 2.35; S, 5.38%. Found: C, 29.89; H, 4.11; N, 2.43; S, 5.41%. **CAS-3**: Yield: 0.48 g, 97%; Anal.: calcd for $C_{84}Zn_6Ca_{5.25}S_6H_{129.5}N_6O_{84}$ (3362.5): C, 30.00; H, 3.88; S, 5.72; N, 2.50%. Found: C, 31.11; H, 4.00; S, 5.81; N, 2.39%.

Kinetic profile of the water dye removal. The adsorption kinetics of organic dyes were investigated under batch (dispersive) conditions using both polycrystalline powdered MOFs **1**–**3** and their corresponding calcium alginate spheres (**CAS-1**, **CAS-2** and **CAS-3**). In a typical experiment, 50 mg of the adsorbent (either powdered MOF or alginate spheres containing the corresponding MOF) were added to 25 mL of a multidye aqueous solution (10 mg L^{-1} of methylene blue, brilliant green, pyronine Y and auramine O) prepared using real water samples collected from the Turia River (Valencia, Spain; 39.5463° N, −0.5477° W). The suspensions were stirred continuously at room temperature.

At selected time intervals (1–120 min), aliquots (1 mL) were withdrawn, centrifuged to remove suspended solids, and diluted to a final volume of 3 mL with deionized water prior to analysis. Dye concentrations were determined by UV–vis spectroscopy using calibration curves obtained from single-component aqueous solutions of each dye at their respective characteristic absorption maxima. All measurements were performed within the linear Beer–Lambert regime (absorbance < 1.0). The measured concentrations were corrected for the dilution factor and used to calculate dye removal efficiencies relative to the initial concentration.

For reusability studies, the adsorption experiments were repeated for up to five consecutive cycles using the same alginate spheres. Between cycles, the spheres were

regenerated by brief immersion in a water/methanol (1:1 v/v) solution for 30 s, followed by direct reuse without further treatment.

Solid-Phase Extraction (SPE) Experiment. Continuous-flow adsorption experiments were performed using a solid-phase extraction (SPE) setup with **CAS-3**. In a typical experiment, 25 mg of the alginate spheres were packed into a polypropylene SPE column (10 mL internal volume) and confined between two porous polyethylene frits (25 μm pore size). A 5 mL multidye aqueous solution containing methylene blue, brilliant green, pyronine Y and auramine O (10 mg L^{-1} each), prepared using real water samples collected from the Turia River (Valencia, Spain), was passed through the column at room temperature.

The eluate was collected and analyzed by UV–vis spectroscopy to determine the residual dye concentrations. Quantitative analysis was carried out using calibration curves obtained from single-component aqueous dye solutions at the characteristic absorption maxima of each dye. Dye removal efficiencies were calculated relative to the initial concentrations.

X-ray Powder Diffraction Measurements. Polycrystalline samples of MOFs **1**–**3** and **CAS-1**, **CAS-2** and **CAS-3** –including both freshly prepared spheres and samples stored in water for three months– were dried in a desiccator for 24 h prior to analysis. The samples were then loaded into 0.5 mm borosilicate glass capillaries, which were mounted and aligned on a Bruker D8 Discover powder diffractometer equipped with Cu Kα radiation (λ = 1.54056 Å). Powder X-ray diffraction patterns were collected at room temperature over a 2θ range of 2–45°. For each sample, five consecutive scans were recorded and merged to obtain a single diffractogram with improved signal-to-noise ratio. Simulated powder diffraction patterns were generated from single-crystal X-ray diffraction data and processed using the Mercury software package (version 4.2.0) provided by the Cambridge Crystallographic Data Centre.[4]

Gas adsorption. The N_2 adsorption-desorption isotherms at 77 K, were carried out on polycrystalline samples of MOFs **1**–**3** and **CAS-1**, **CAS-2** and **CAS-3** with a BELSORP-miniX instrument. Samples were first activated with methanol and then evacuated at 348 K during 19 hours under 10^{-6} Torr prior to their analysis.

UV-vis spectroscopy. UV-vis spectra were recorded, at room temperature, with a Jasco V 670 spectrometer.

Microscopy measurements. Scanning Electron Microscopy coupled with Energy Dispersive X–ray (SEM/EDX) was carried out with a XL 30 ESEM (PHILIPS) microscope equipped with a home–made EDX energy dispersive X–ray detector.

Table S1. Selected data for the dye adsorption,[a] obtained from the UV-vis measurements, of 25 mL of a multidye solution with real water samples from Turia river (Valencia, Spain) spiked with 10 ppm of AO, BG, MB and PY, after soaking 50 mg of MOFs **1**-**3**.

Time (min)	AO (%)	BG (%)	MB (%)	PY (%)
		**MOF 1**		
1	9.4	35.7	66.8	48.3
5	25.4	54.3	77.5	62.2
15	43.6	65.4	84.5	71.6
30	59.9	75.2	87.8	78.3
60	76.7	85.7	91.4	87.7
120	88.0	92.3	95.3	92.5
		**MOF 2**		
1	78.2	76.1	71.1	63.1
5	87.8	89.1	88.3	83.7
15	96.3	97.0	96.6	97.4
30	98.6	98.9	98.8	97.8
60	99.6	99.6	99.4	98.3
120	99.8	99.8	99.9	99.2
		**MOF 3**		
1	89.9	88.0	90.8	88.5
5	95.8	95.9	96.6	93.9
15	99.3	98.7	99.0	98.9
30	99.3	99.2	99.4	99.2
60	99.8	99.8	99.3	99.3
120	99.7	99.9	99.5	99.7

[a] Dye uptake (in %) extracted from the UV-vis measurements (see experimental section).

Table S2. Selected data for the dye adsorption,[a] obtained from the UV-vis measurements, of a multidye solution with real water samples from Turia river (Valencia, Spain) spiked with 10 ppm of AO, BG, MB and PY, after soaking 25 mg of MOF **1** + 25 mg of MOF **2**.

Time (min)	AO (%)	BG (%)	MB (%)	PY (%)
1	61.8	72.8	71.6	70.1
5	78.0	80.7	78.7	79.5
15	84.8	86.6	83.6	83.7
30	89.1	91.4	88.6	87.7
60	93.2	95.4	92.5	93.2
120	96.9	97.9	95.4	96.1

[a] Dye uptake (in %) extracted from the UV-vis measurements (see experimental section).

Table S3. Selected data for the dye adsorption,[a] obtained from the UV-vis measurements, of a multidye solution with real water samples from Turia river (Valencia, Spain) spiked with 10 ppm of AO, BG, MB and PY, after soaking 50 mg of calcium alginate spheres of MOF **1**.

Time (min)	AO (%)	BG (%)	MB (%)	PY (%)
		**1st cycle**		
1	30.0	31.2	28.4	29.9
5	45.6	43.2	44.2	47.8
15	62.2	66.7	58.2	67.2
30	76.5	78.4	77.1	79.4
60	86.1	85.3	84.1	85.8
120	90.6	91.8	92.1	91.2
		**2nd cycle**		
1	31.8	28.3	30.8	29.6
5	47.2	46.3	48.1	45.9
15	65.0	66.3	69.4	62.7
30	74.3	73.9	76.4	78.2
60	85.2	84.9	83.9	86.3
120	89.4	91.3	90.2	90.9
		**3rd cycle**		
1	25.5	28.3	27.4	28.5
5	43.8	44.6	45.1	46.9
15	64.3	66.2	63.0	67.4
30	72.8	75.3	74.3	77.4
60	83.8	86.2	85.8	87.4
120	86.9	89.9	92.3	91.8
		**4th cycle**		
1	28.9	30.2	28.4	29.5
5	46.6	45.2	47.4	44.2
15	66.7	65.1	67.4	63.9
30	76.9	77.4	75.9	72.7
60	84.9	85.2	86.4	83.8
120	87.1	90.0	91.3	88.5
		**5th cycle**		
1	25.5	27.3	28.4	30.1
5	46.2	44.2	43.1	48.4
15	66.2	64.0	67.4	69.5
30	77.9	72.6	77.4	77.8
60	83.5	84.6	86.9	87.4
120	88.8	87.5	92.3	91.6

[a] Dye uptake (in %) extracted from the UV-vis measurements (see experimental section).

Table S4. Selected data for the dye adsorption,[a] obtained from the UV-vis measurements, of a multidye solution with real water samples from Turia river (Valencia, Spain) spiked with 10 ppm of AO, BG, MB and PY, after soaking 50 mg of calcium alginate spheres of MOF **2**.

Time (min)	AO (%)	PY (%)	BG (%)	MB (%)
		**1st cycle**		
1	65.5	64.7	64.2	66.9
5	80.5	81.2	78.6	79.8
15	92.4	91.7	92.2	90.5
30	98.7	97.4	97.7	98.0
60	99.7	99.0	99.3	99.6
120	99.6	99.8	99.5	99.4
		**2nd cycle**		
1	66.6	64.2	66.8	63.7
5	78.8	80.2	81.0	79.3
15	93.4	92.2	94.4	91.9
30	97.9	98.3	99.0	98.3
60	99.3	98.7	99.3	99.6
120	99.2	99.9	99.9	99.6
		**3rd cycle**		
1	64.1	65.6	66.8	63.8
5	80.3	79.5	78.9	81.2
15	94.3	92.9	93.0	92.9
30	98.1	99.4	99.4	99.1
60	99.8	99.1	99.5	99.6
120	99.9	99.3	99.8	99.5
		**4th cycle**		
1	63.5	63.8	66.1	64.4
5	77.3	79.8	82.5	80.1
15	93.9	92.8	94.7	95.1
30	99.3	99.2	99.1	99.3
60	99.4	99.4	99.2	99.6
120	99.5	99.6	99.9	99.7
		**5th cycle**		
1	60.3	61.8	62.9	64.7
5	79.3	82.1	81.6	82.0
15	94.1	93.9	95.2	95.0
30	98.2	99.2	98.7	98.4
60	99.3	99.2	99.4	99.3
120	99.6	99.3	99.9	99.4

[a] Dye uptake (in %) extracted from the UV-vis measurements (see experimental section).

Table S5. Selected data for the dye adsorption,[a] obtained from the UV-vis measurements, of a multidye solution with real water samples from Turia river (Valencia, Spain) spiked with 10 ppm of AO, BG, MB and PY, after soaking 50 mg of calcium alginate spheres of MOF **3**.

Time (min)	AO (%)	PY (%)	BG (%)	MB (%)
		**1st cycle**		
1	79.3	80.5	79.1	80.6
5	91.5	90.7	90.3	92.3
15	99.2	98.7	98.2	99.0
30	99.7	99.4	99.7	99.0
60	99.7	99.0	99.3	99.6
120	99.6	99.8	99.5	99.4
		**2nd cycle**		
1	82.6	83.0	81.8	84.8
5	94.9	92.0	93.2	94.0
15	99.4	99.0	99.0	99.4
30	99.0	99.1	99.7	99.3
60	99.0	99.7	99.6	99.3
120	99.4	99.3	99.9	99.7
		**3rd cycle**		
1	78.4	80.2	78.8	80.0
5	92.2	91.3	93.9	93.5
15	99.3	99.2	98.9	98.9
30	99.6	99.2	99.4	99.0
60	99.8	99.8	99.7	99.6
120	99.3	99.1	99.5	99.4
		**4th cycle**		
1	81.8	80.8	79.5	82.1
5	91.8	92.3	93.5	93.8
15	98.9	98.9	99.2	98.4
30	99.3	99.6	99.1	99.0
60	99.3	99.4	99.3	99.1
120	99.2	99.6	99.2	99.2
		**5th cycle**		
1	79.3	81.2	77.9	76.2
5	93.3	92.4	92.2	93.0
15	99.2	98.4	99.4	98.0
30	99.3	99.9	99.1	99.4
60	99.2	99.9	99.4	99.0
120	99.4	99.3	99.5	99.4

[a] Dye uptake (in %) extracted from the UV-vis measurements (see experimental section).

Table S6. Selected data for the dye adsorption,[a] obtained from the UV-vis measurements, of a multidye solution with real water samples from Turia river (Valencia, Spain) spiked with 10 ppm of AO, BG, MB and PY, after soaking 50 mg of pristine calcium alginate spheres.

Time (min)	AO (%)	BG (%)	MB (%)	PY (%)
1	4.6	5.9	4.4	5.3
5	5.4	7.2	6.1	5.9
15	6.1	7.7	6.6	8.9
30	6.8	8.3	7.2	9.7
60	7.6	9.4	7.9	10.8
120	10.1	9.7	8.7	11.8

[a] Dye uptake (in %) extracted from the UV-vis measurements (see experimental section).

Table S7. Selected dye recovery data,a obtained from UV–vis analysis of the water/methanol (1:1 v/v) regeneration solutions after 30 s treatment of dye-loaded **CAS-1**, **CAS-2** and **CAS-3**. The spheres had been previously exposed to a multidye solution prepared using real water samples collected from the Turia River (Valencia, Spain) and spiked with 10 mg L^{-1} of AO, BG, MB and PY.

Composite	AO (%)	BG (%)	MB (%)	PY (%)
**CAS-1**	46.3	55.8	52.4	54.3
**CAS-2**	53.2	53.8	59.2	49.8
**CAS-3**	54.2	58.7	48.2	59.2

Table S8. Selected data for the dye adsorption,[a] at pH = 4 and 10, obtained from the UV-vis measurements, of a multidye solution with real water samples from Turia river (Valencia, Spain) spiked with 10 ppm of AO, BG, MB and PY, after soaking 50 mg of **CAS-3**.

Time (min)	AO (%)	BG (%)	MB (%)	PY (%)
		**pH = 4**		
1	70.5	71.1	79.3	87.9
5	86.8	87.7	90.3	95.3
15	96.2	95.3	97.3	98.9
30	99.4	99.8	99.3	99.8
60	99.8	99.9	99.9	99.9
120	99.9	99.9	99.8	99.7
		**pH = 10**		
1	79.8	80.9	77.9	75.1
5	87.3	87.4	85.2	87.3
15	97.3	96.7	97.4	95.4
30	99.5	99.0	98.8	99.8
60	99.9	99.7	99.3	99.6
120	99.8	99.7	99.8	99.8

[a] Dye uptake (in %) extracted from the UV-vis measurements (see experimental section).

Table S9. Continuous-flow removal efficiency of MTV-MOF **3** calmium alginate spheres (50 mg) towards 25 mL of a multidye solution with real water samples from Turia river (Valencia, Spain) spiked with 10 ppm of AO, BG, MB and PY.

AO (%)	BG (%)	MB (%)	PY (%)
99.7	99,7	99,8	99,3

[a] Dye uptake (in %) extracted from the UV-vis measurements (see experimental section).

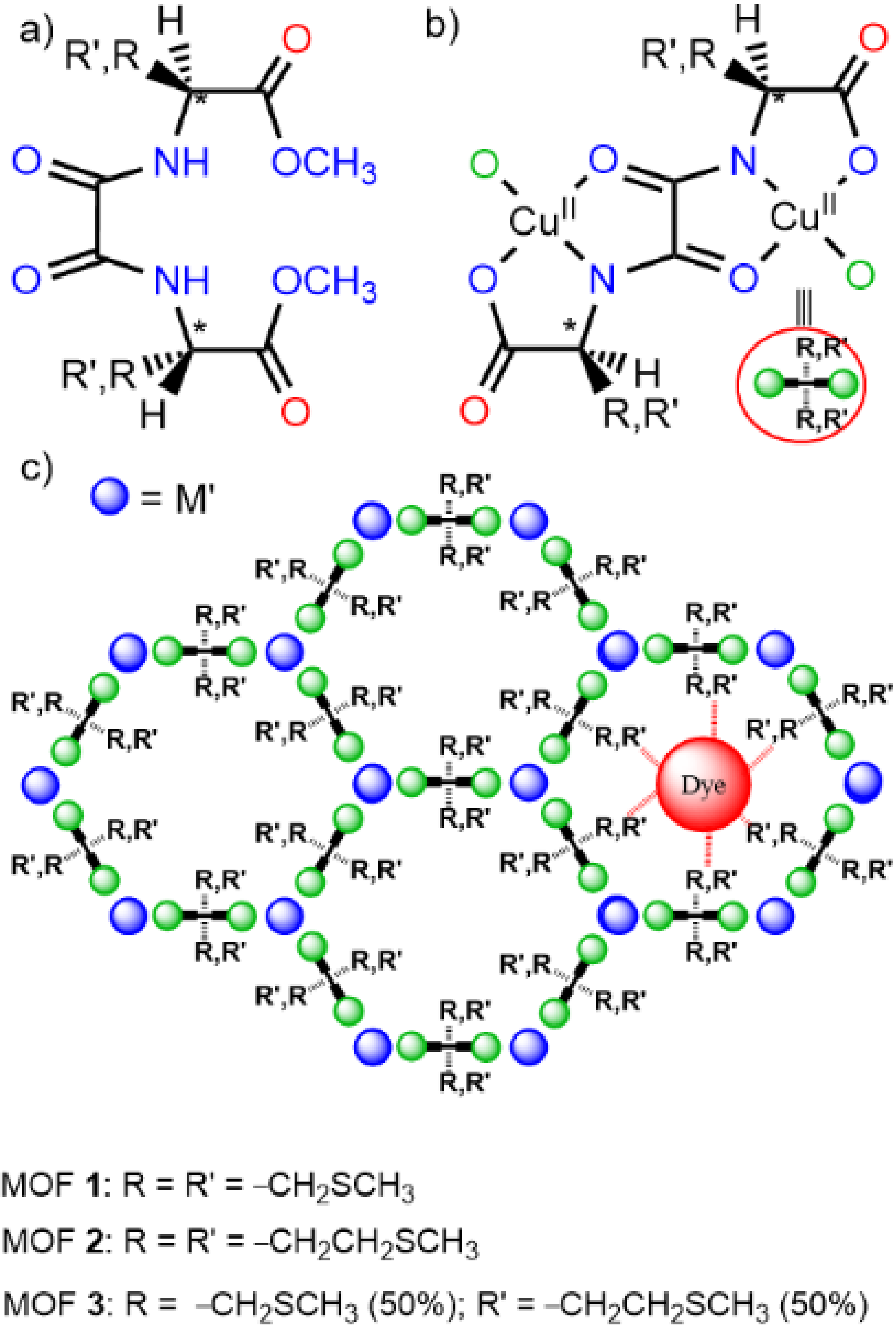

Scheme S1. Chemical structures of the amino acid-based oxamidato ligands (a) and dinuclear zinc(II) precursor complexes (b), as well as a schematic representation of the MOF hexagonal network emphasizing the position of the amino acid residues (R = R' = $–CH_2SCH_3$ MOF **1**; R = R' = $–CH_2CH_2SCH_3$ MOF **2**; R = $–CH_2SCH_3$ and R' = $CH_2CH_2SCH_3$ MTV-MOF **3**). Red surface represents the organic dyes embedded within the MOFs' channels interacting with amino acid residues decorating the pores.

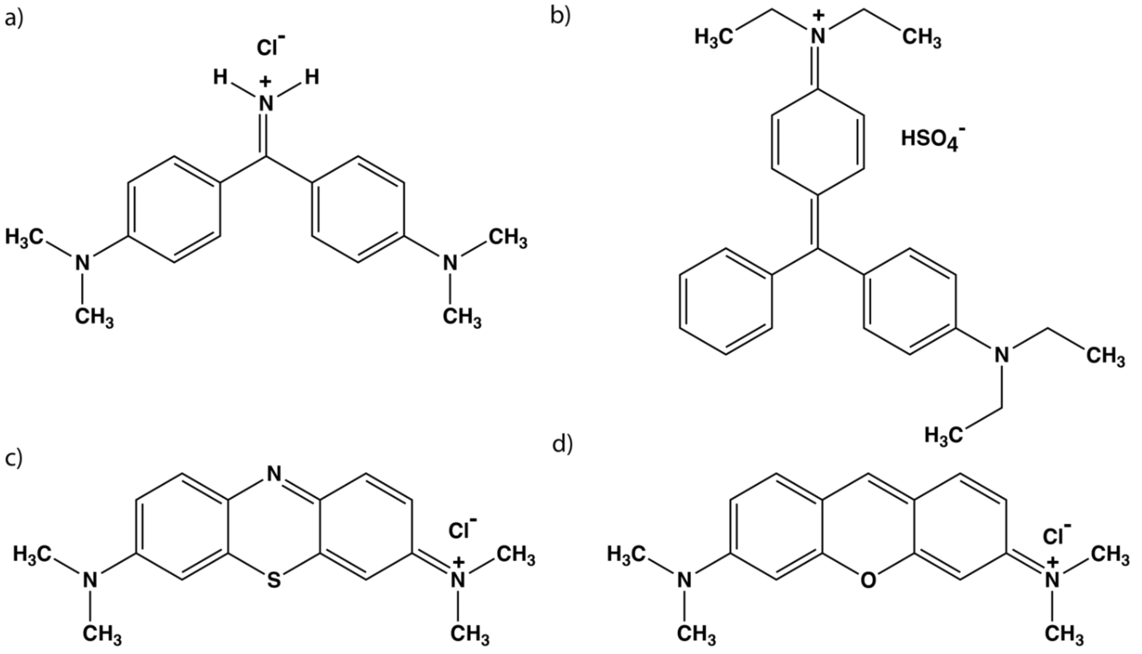

Scheme S2. Chemical structures of Auramine O (a), Brilliant Green (b), Methylene Blue (c) and Pyronin Y (d).

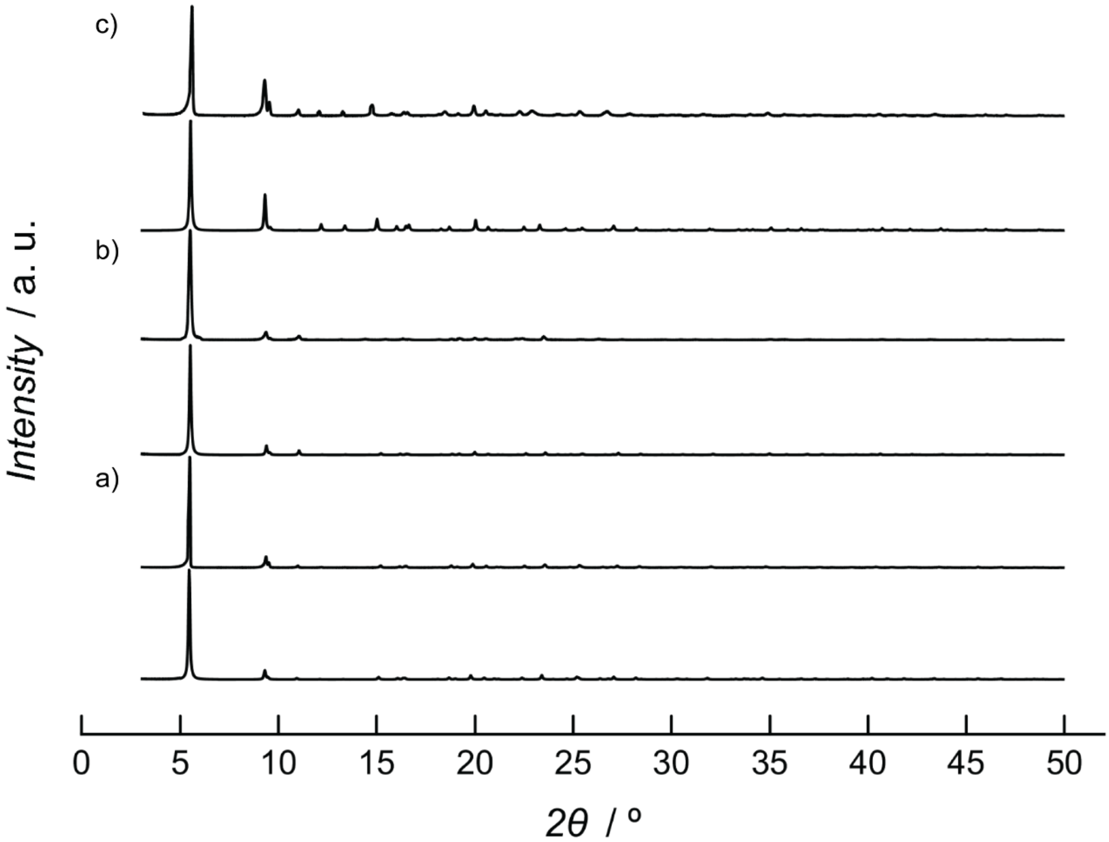

Fig. S1. Theoretical (bottom) and experimental (top) PXRD pattern profiles of **1** (a), **2** (b) and **3** (c) in the form of polycrystalline powders in the 2θ range 3.0–50.0.

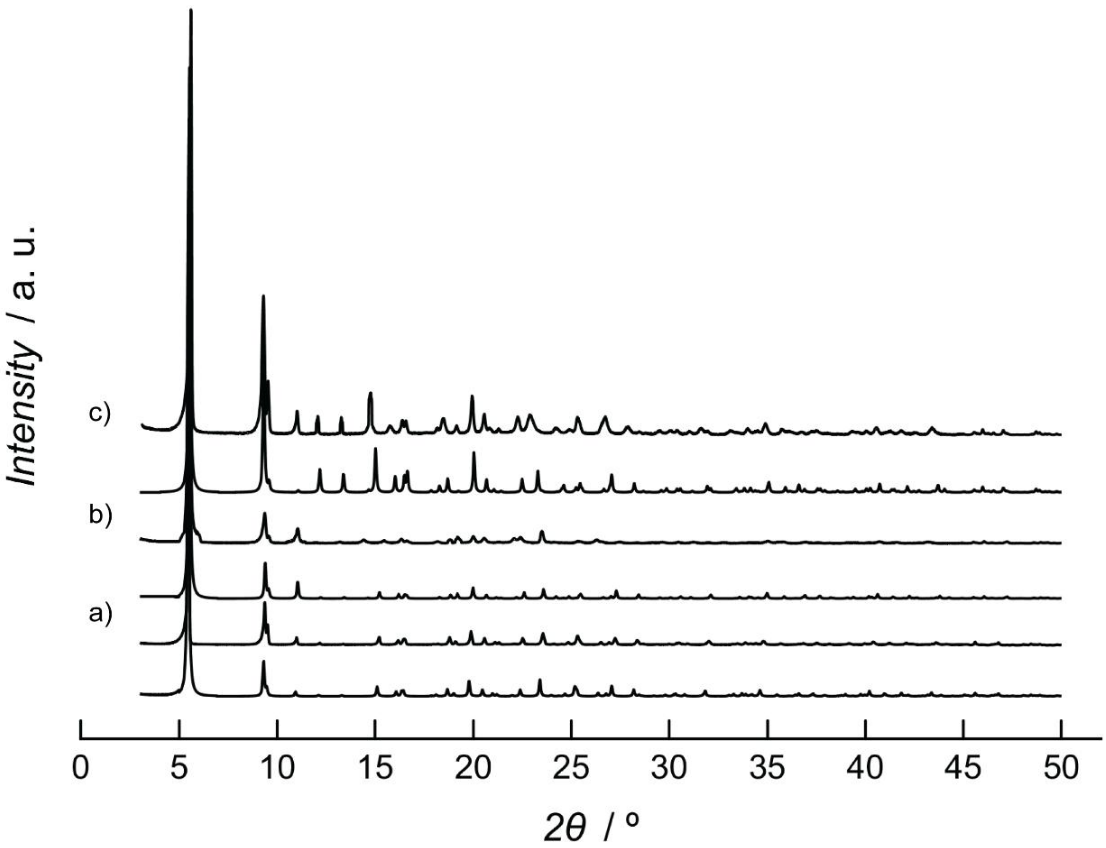

Fig. S2. Superimposed theoretical (bottom) and experimental (top) PXRD pattern profiles of **1** (a), **2** (b) and **3** (c) in the form of polycrystalline powders in the 2θ range 3.0–50.0.

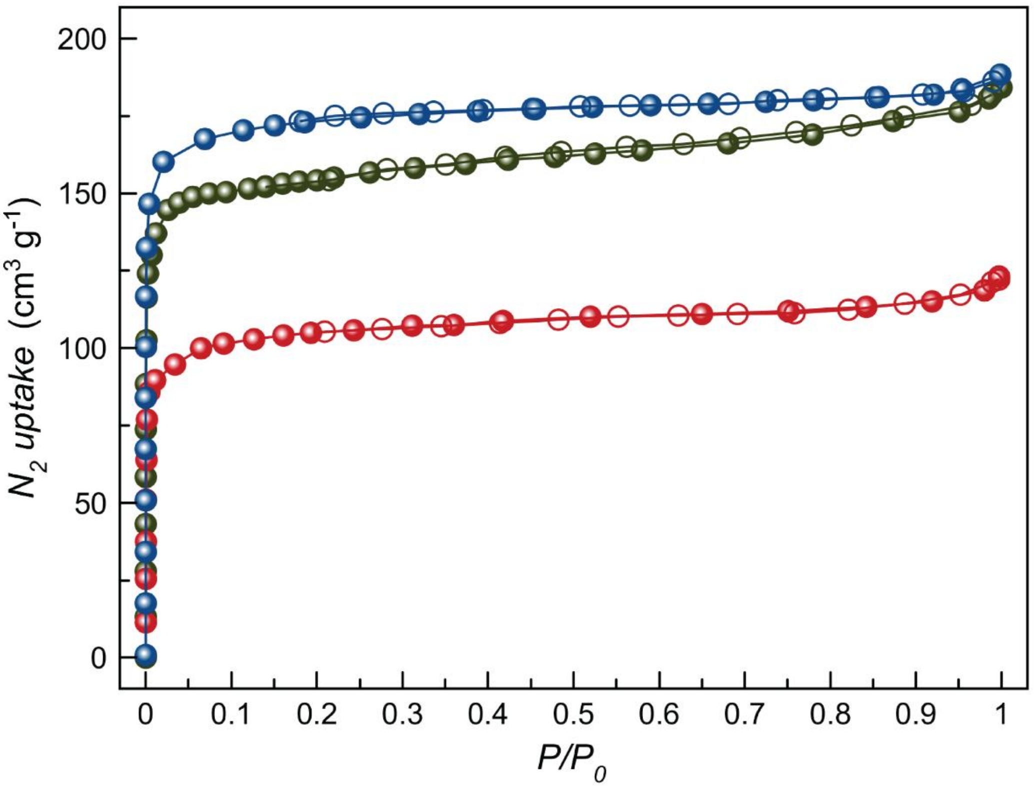

Fig. S3. N_2 (77 K) adsorption isotherm for the activated compounds **1** (blue), **2** (red) and **3** (green). Filled and empty symbols indicate the adsorption and desorption isotherms, respectively. The samples were activated at 100ºC under reduced pressure for 19 h prior to carry out the sorption measurements.

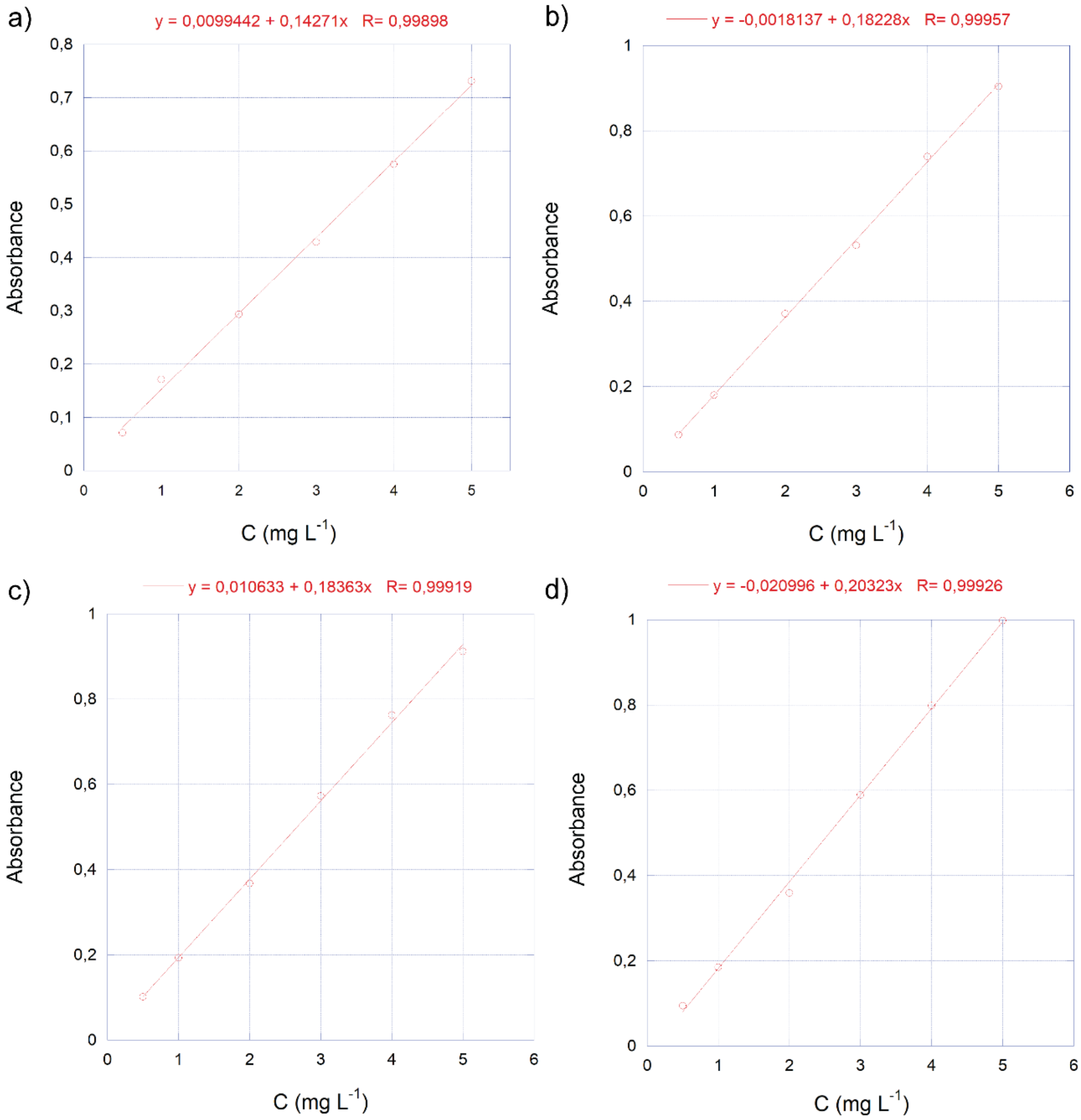

Fig. S4. UV–vis calibration curves for methylene blue (MB), brilliant green (BG), pyronine Y (PY) and auramine O (AO) obtained from single-component aqueous solutions at their characteristic absorption maxima. Linear regression fits and corresponding equations were used for quantitative determination of dye concentrations in the multidye adsorption experiments.

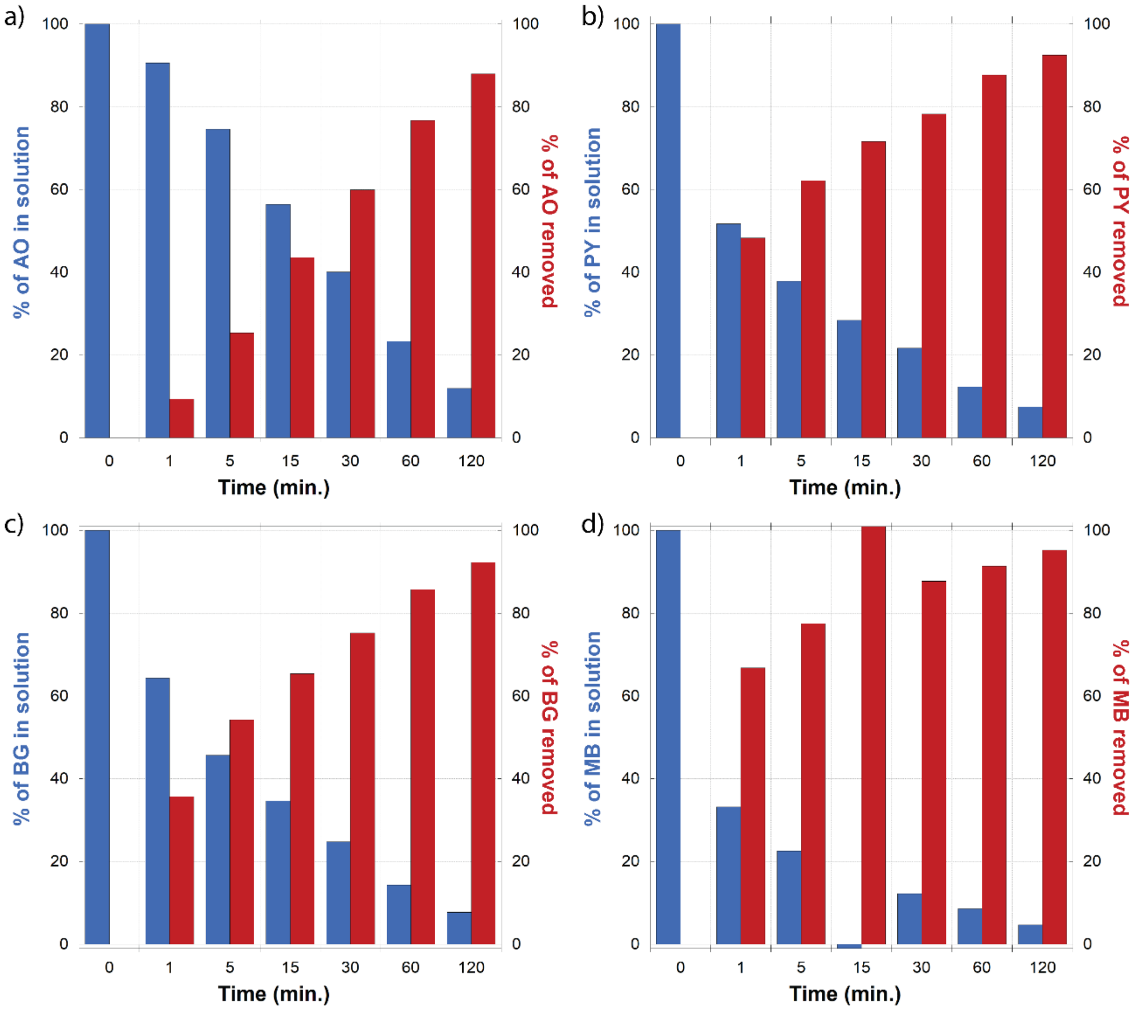

Fig. S5. Evolution with time of the adsorption of AO (a), BG (b), MB (c) and PY (d) in 10 ppm solutions using real water samples from Turia river (50 mg of MOF **1** as adsorbent), Blue bars represent the % of each dye within the solution and red bars represent the % of the dye removed from the solution.

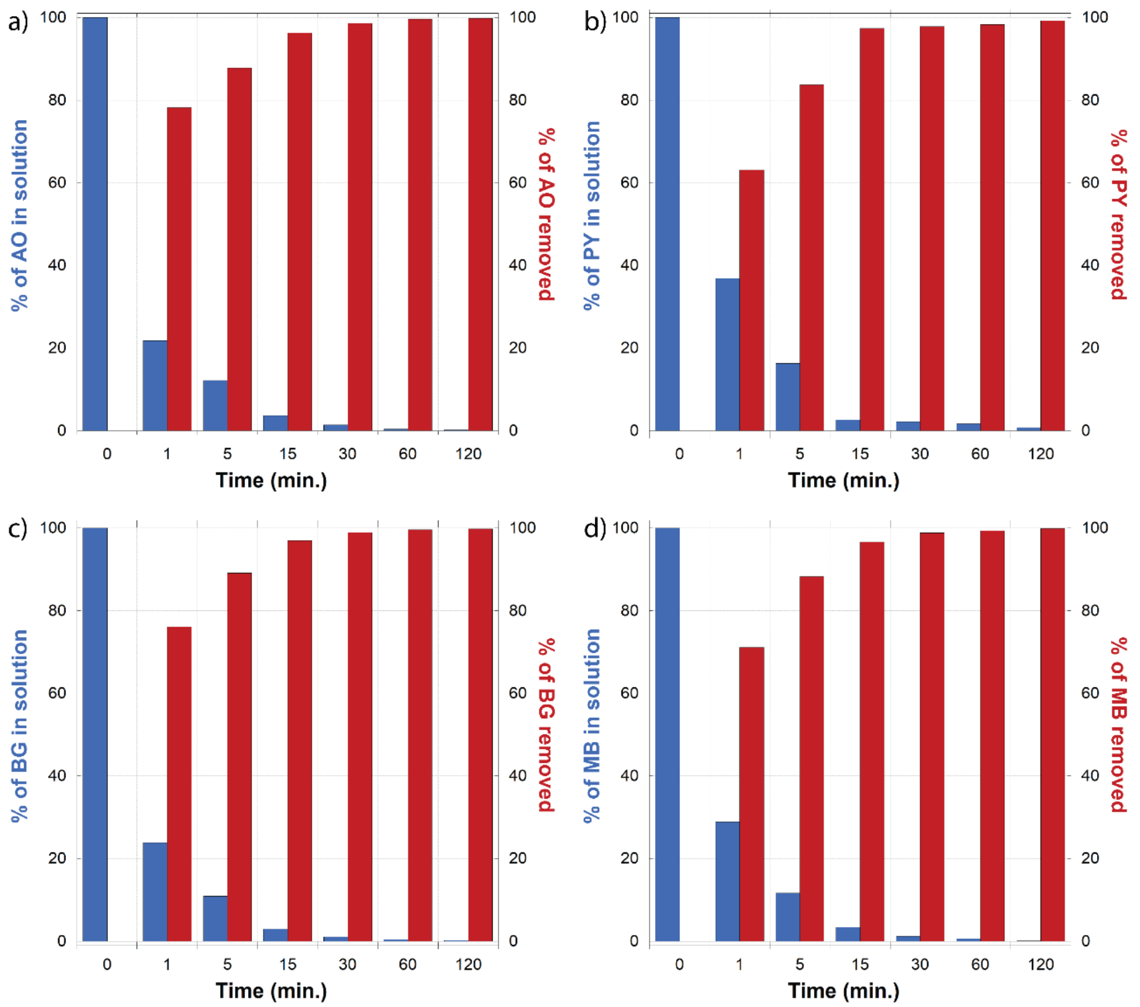

Fig. S6. Evolution with time of the adsorption of AO (a), BG (b), MB (c) and PY (d) in 10 ppm solutions using real water samples from Turia river (50 mg of MOF **2** as adsorbent), Blue bars represent the % of each dye within the solution and red bars represent the % of the dye removed from the solution.

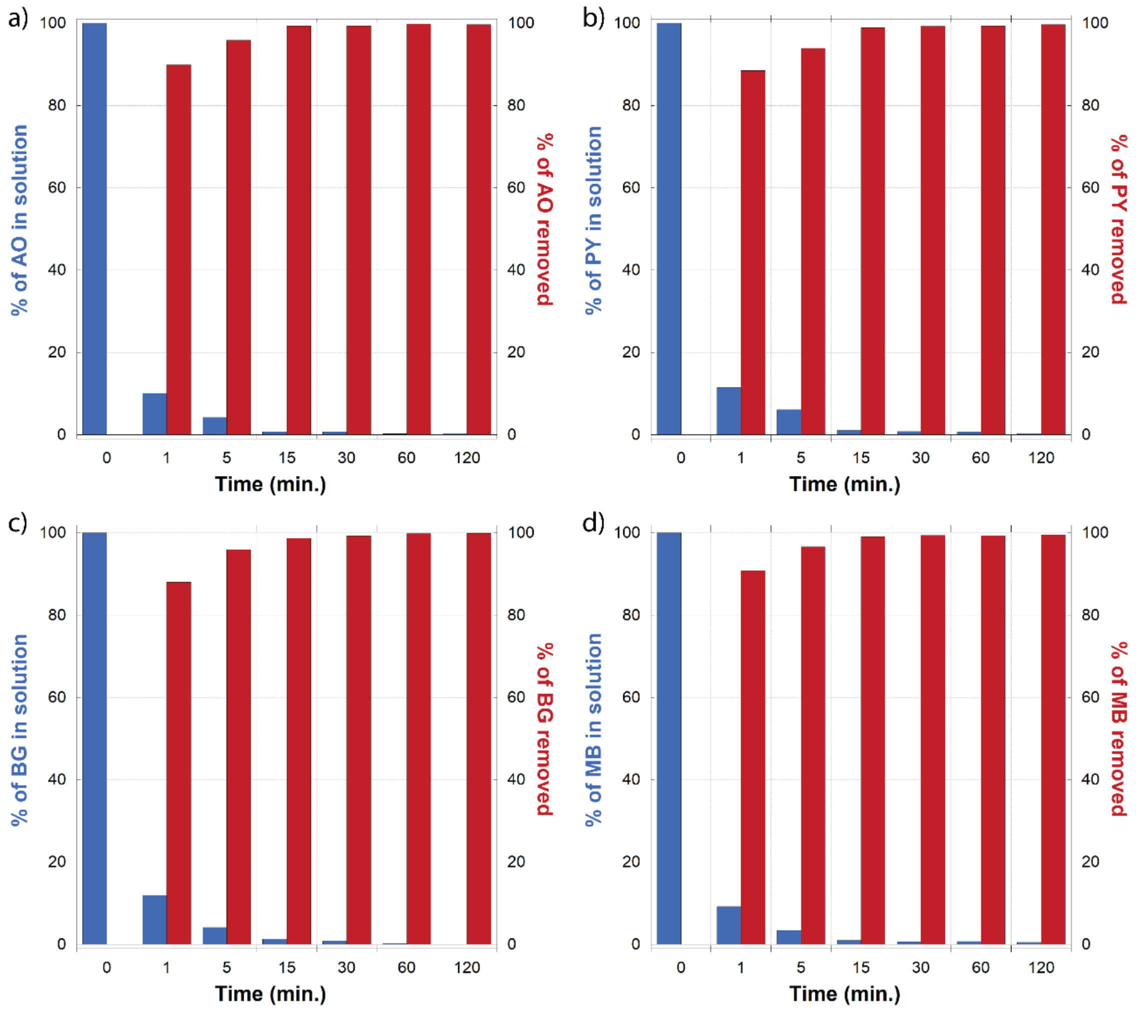

Fig. S7. Evolution with time of the adsorption of AO (a), BG (b), MB (c) and PY (d) in 10 ppm solutions using real water samples from Turia river (50 mg of MTV-MOF **3** as adsorbent), Blue bars represent the % of each dye within the solution and red bars represent the % of the dye removed from the solution.

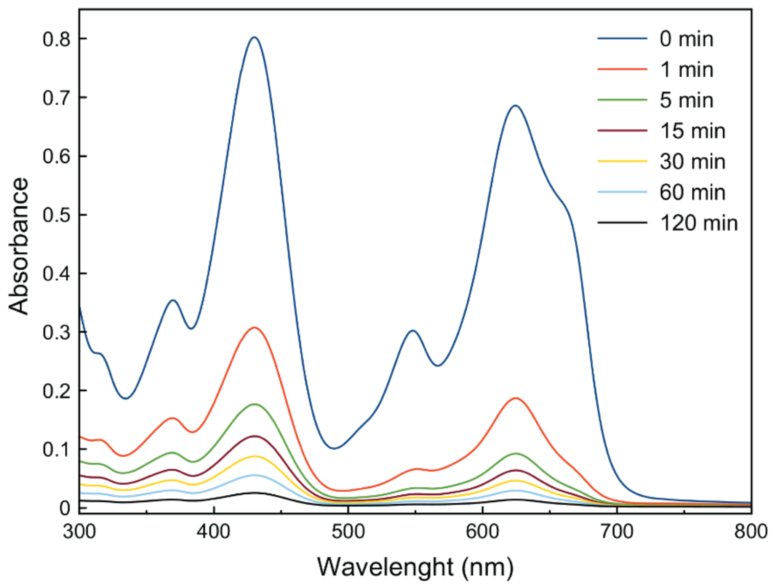

Fig. S8. Evolution with time of the UV-Vis absorption spectra of a multidye solution containing 10 ppm solutions of Auramine O, Brilliant green, Methylene blue and Pyronin Y in real water samples from Turia river in the presence of 25 mg of MOF **1** + 25 mg of MOF **2**.

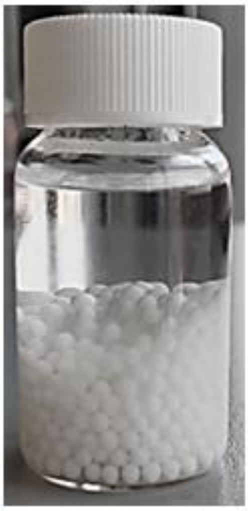

Fig. S9. Photograph of the "as-synthesized" calcium alginate spheres containing MTV-MOF **3** (**CAS-3**) stored in water.

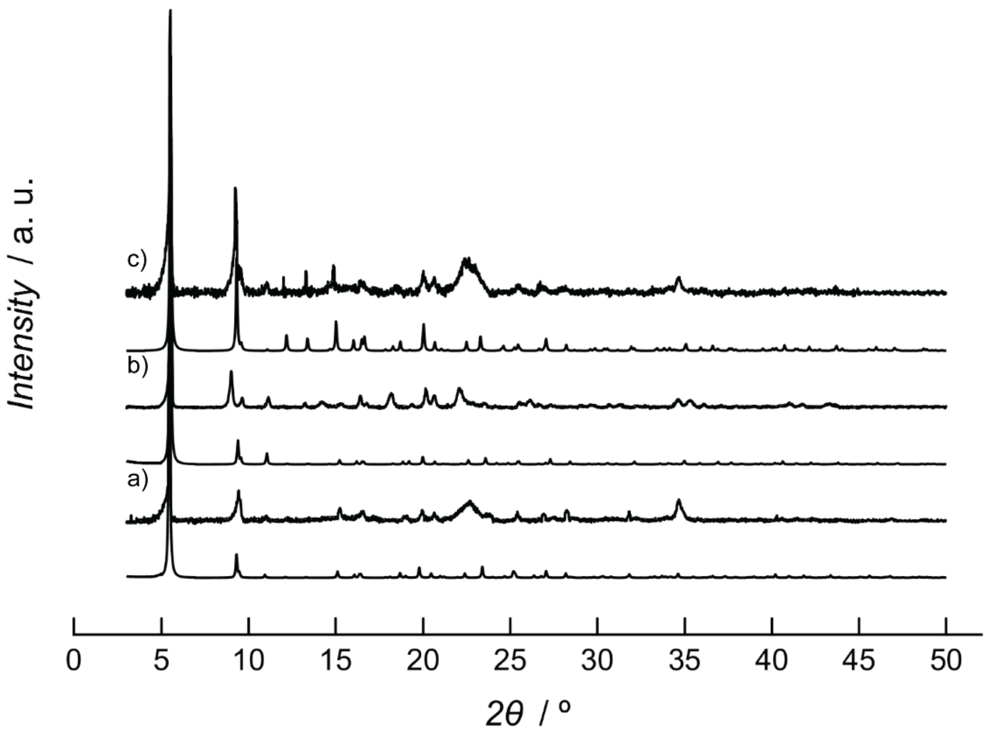

Fig. S10. Theoretical (bottom) and experimental (top) PXRD pattern profiles of MOFs **1** (a), **2** (b) and **3** (c), embedded at calcium alginate spheres (**CAS-1**, **CAS-2** and **CAS-3**), in the 2θ range 3.0–50.0.

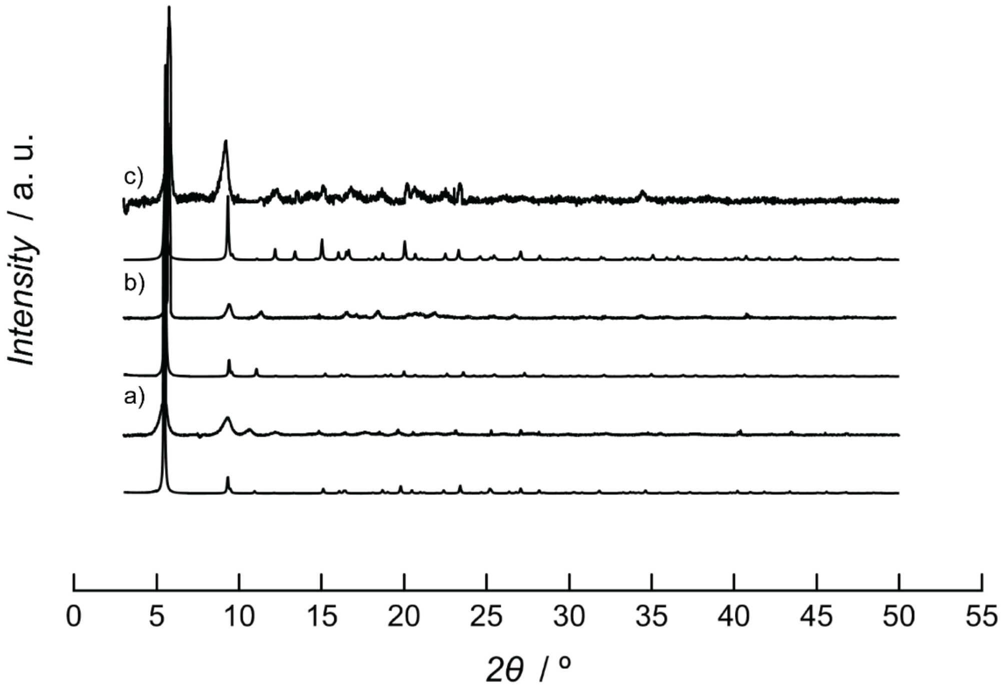

Fig. S11. Theoretical (bottom) and experimental (top) PXRD pattern profiles of MOFs **1** (a), **2** (b) and **3** (c), embedded at calcium alginate spheres (**CAS-1**, **CAS-2** and **CAS-3**), in the 2θ range 3.0–50.0, after 3 months stored in water.

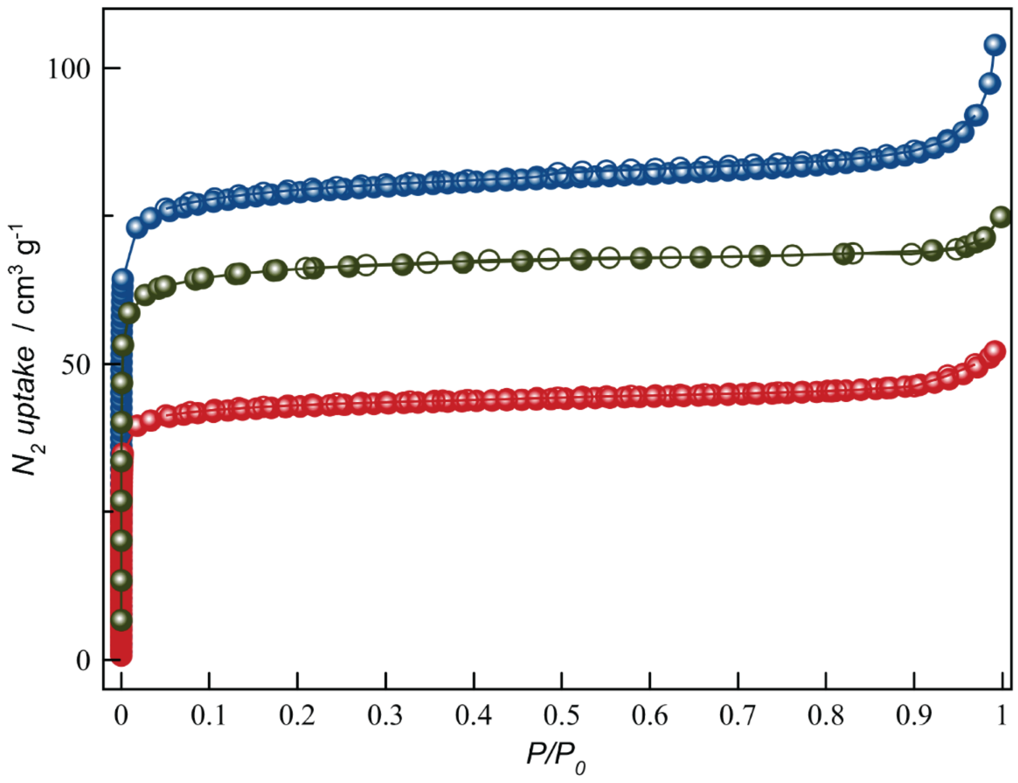

Fig. S12. N_2 (77 K) adsorption isotherms for **CAS-1** (blue), **CAS-2** (red) and **CAS-3** (green). Filled and empty symbols indicate the adsorption and desorption isotherms, respectively. The samples were activated at 100ºC under reduced pressure for 19 h prior to carry out the sorption measurements.

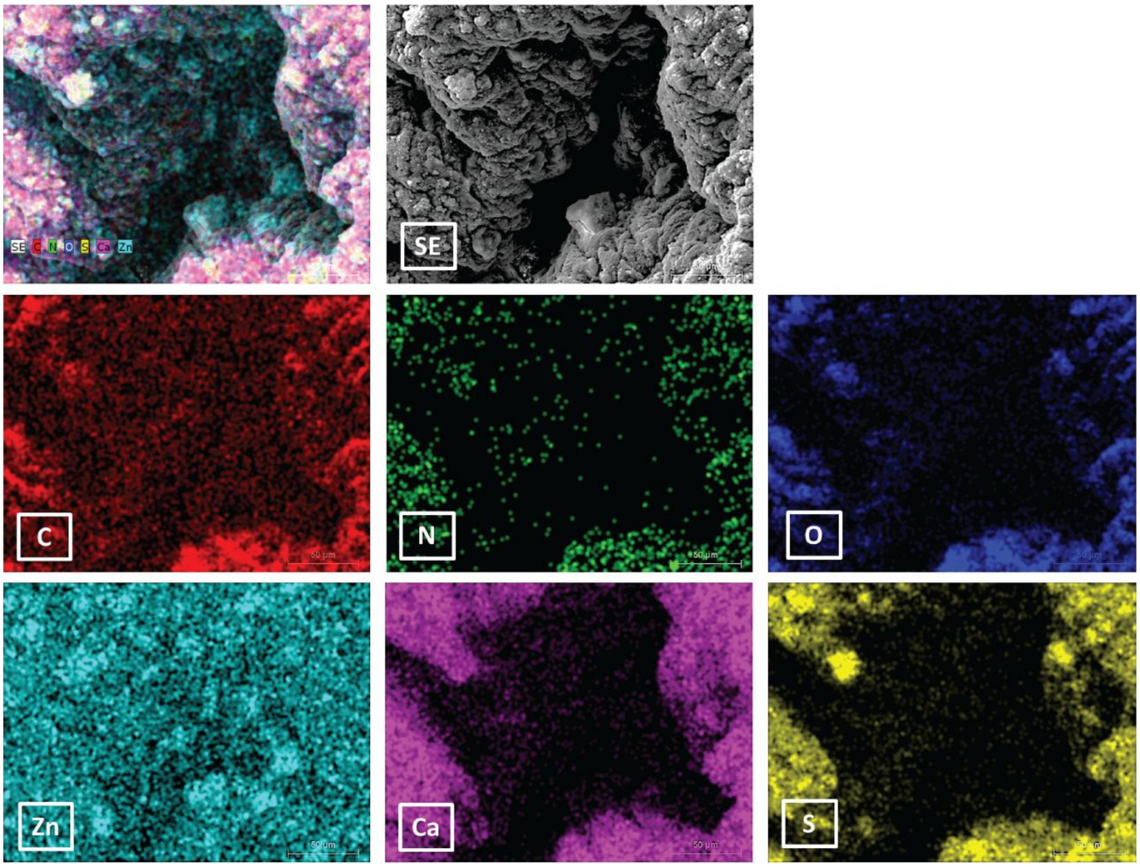

Fig. S13. (a) SEM image of a calcium alginate sphere of MOF **1** (**CAS-1**) and the corresponding EDX elemental mapping for C, N, O, Zn, Ca and S elements.

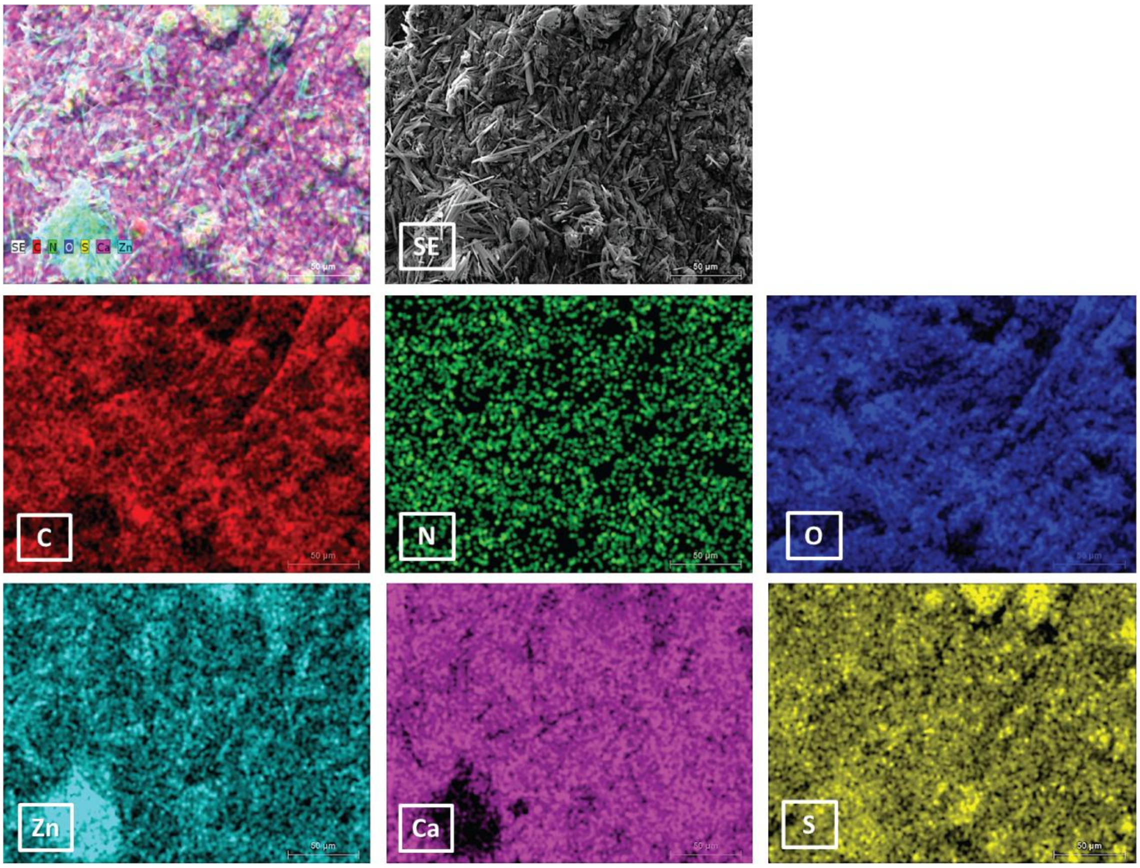

Fig. S14. (a) SEM image of a calcium alginate sphere of MOF **2** (**CAS-2**) and the corresponding EDX elemental mapping for C, N, O, Zn, Ca and S elements.

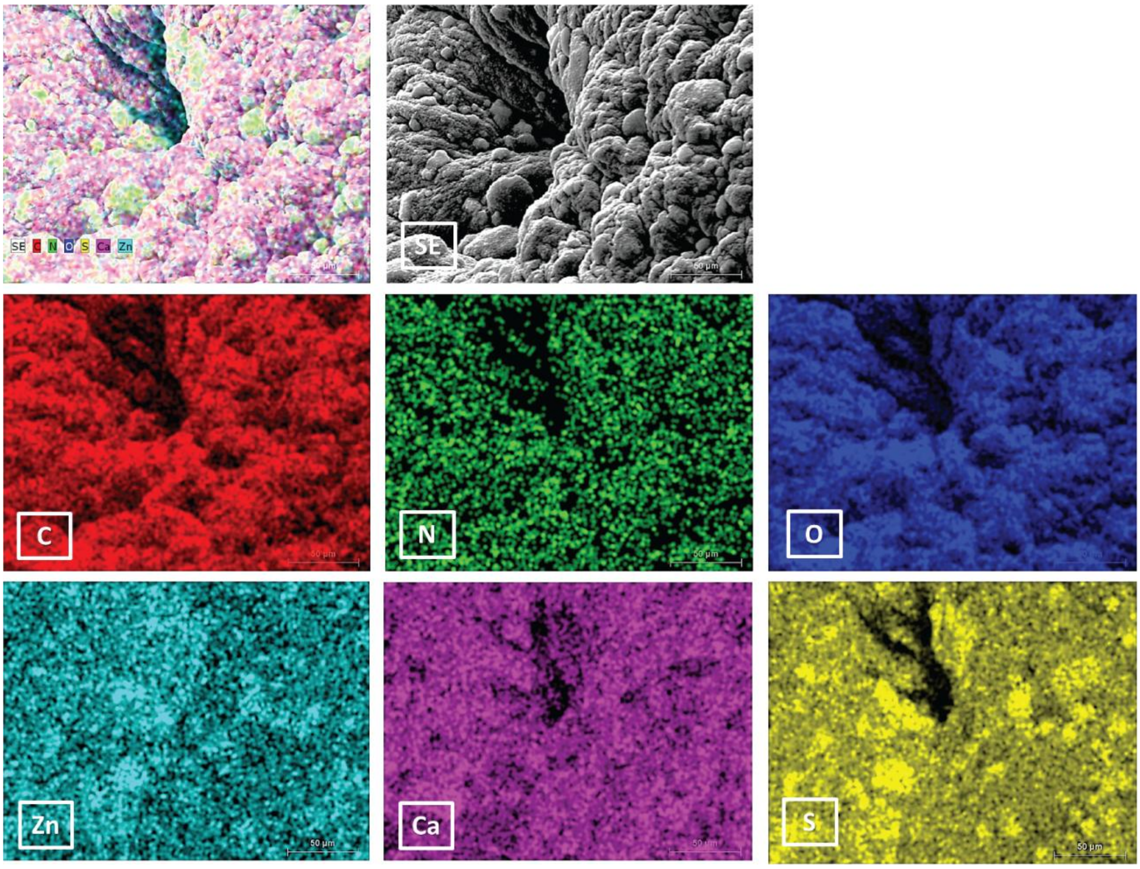

Fig. S15. (a) SEM image of a calcium alginate sphere of MTV-MOF **3** (**CAS-3**) and the corresponding EDX elemental mapping for C, N, O, Zn, Ca and S elements.

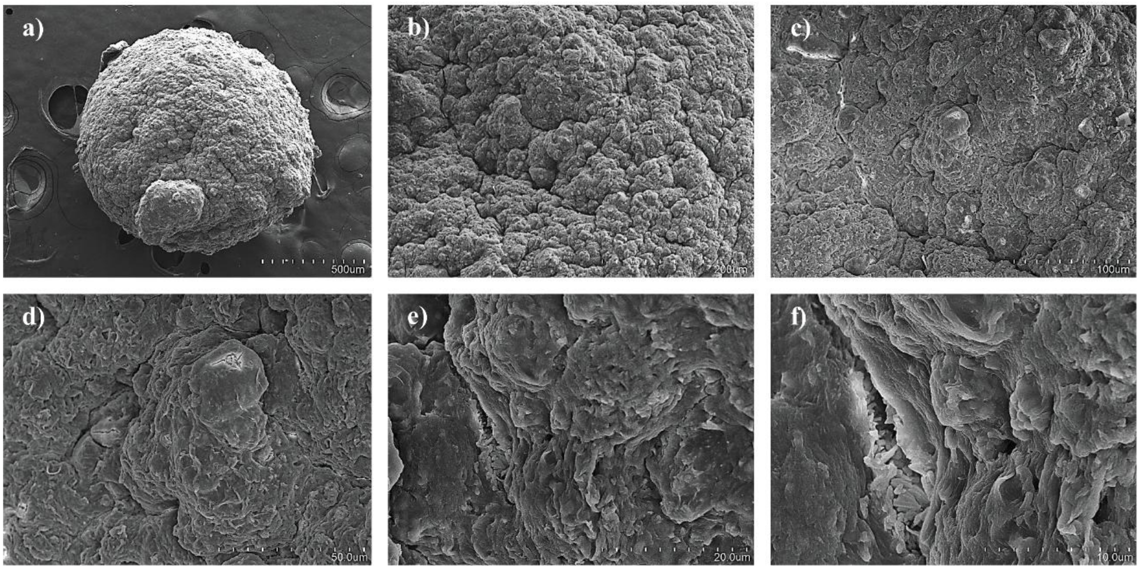

Fig. S16. (a) SEM image of a calcium alginate sphere of MOF **2** (**CAS-2**) and (b-f) different zoomed areas of the sphere.

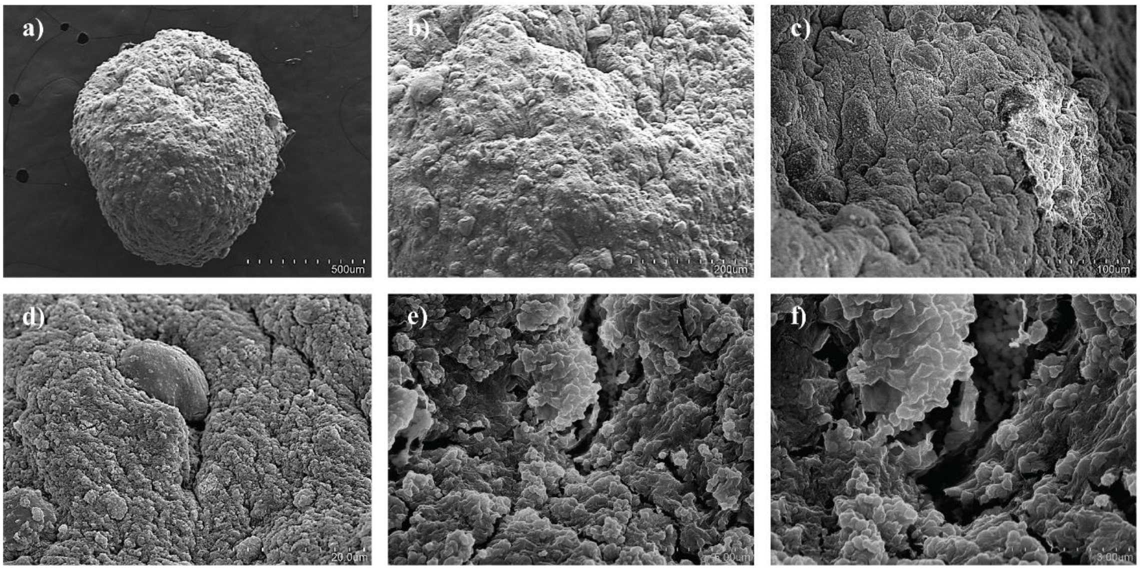

Fig. S17. (a) SEM image of a calcium alginate sphere of MOF **3** (**CAS-3**) and (b-f) different zoomed areas of the sphere.

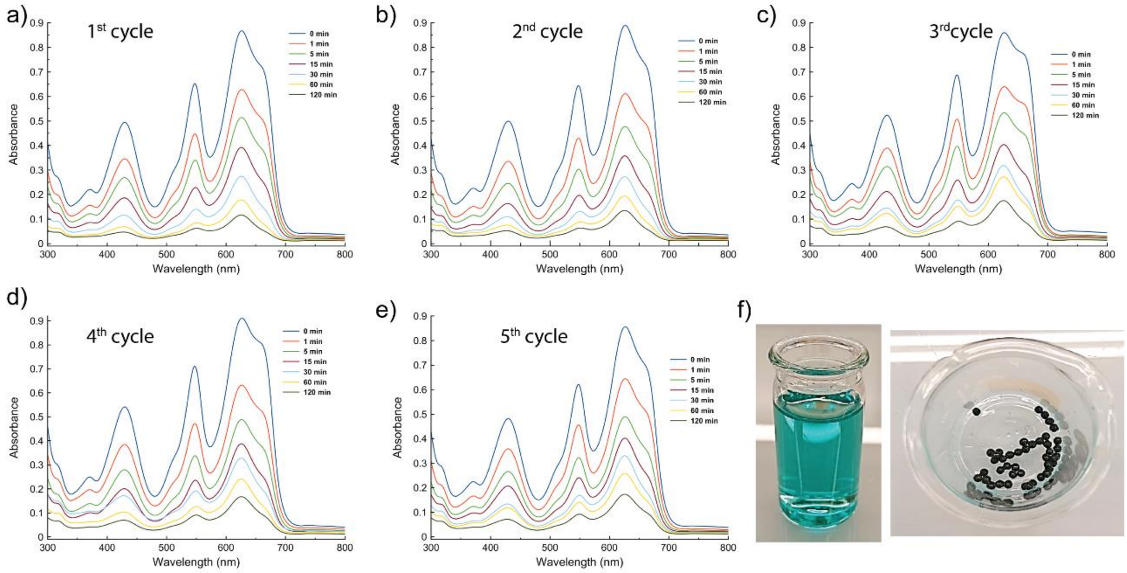

Fig. S18. Time evolution of the UV–Vis absorption spectra of a multidye solution containing 10 mg L^{-1} each of Auramine O, Brilliant Green, Methylene Blue, and Pyronin Y in real water samples from the Turia River, in the presence of 50 mg of **CAS-1**, over five consecutive cycles (a–e). (f) Photograph of the multidye solution before the capture experiments (left) and image of spheres of **CAS-1** together with the resulting colourless solution after 120 minutes in the fifth capture cycle.

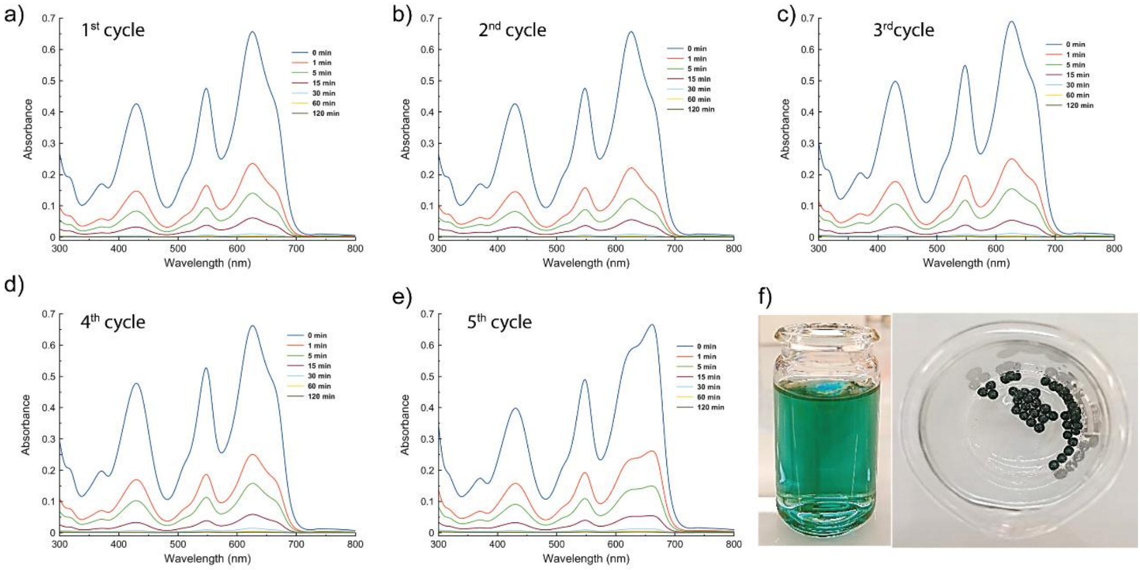

Fig. S19. Time evolution of the UV–Vis absorption spectra of a multidye solution containing 10 mg L^{-1} each of Auramine O, Brilliant Green, Methylene Blue, and Pyronin Y in real water samples from the Turia River, in the presence of 50 mg of **CAS-2**, over five consecutive cycles (a–e). (f) Photograph of the multidye solution before the capture experiments (left) and image of spheres of **CAS-2** together with the resulting colourless solution after 120 minutes in the fifth capture cycle.

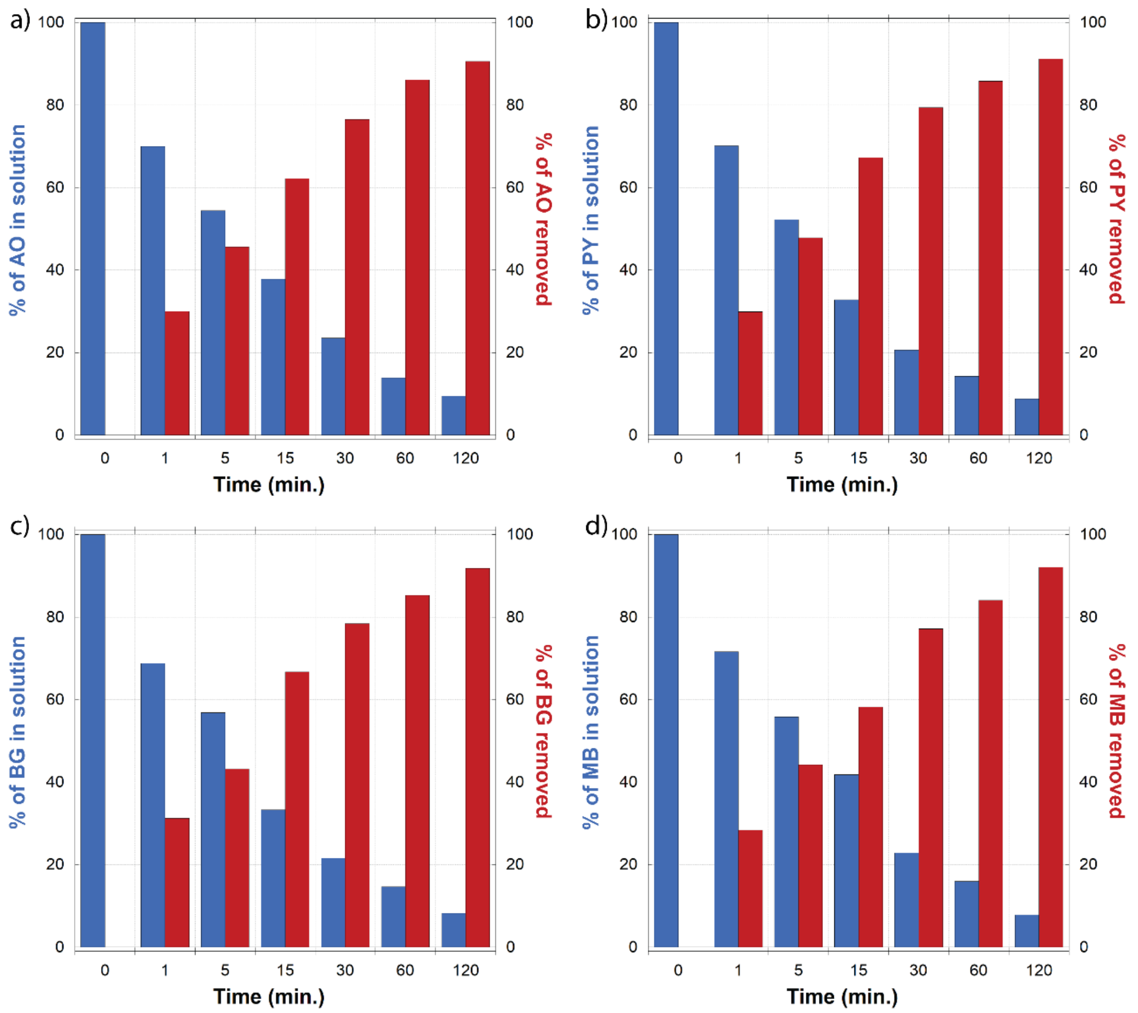

Fig. S20. Time-dependent adsorption of auramine O (AO, a), brilliant green (BG, b), methylene blue (MB, c) and pyronine Y (PY, d) from real water samples collected from the Turia River (Valencia, Spain) using 50 mg of **CAS-1** as the adsorbent during the first adsorption cycle. The initial dye concentration was 10 mg L^{-1} for each dye. Blue bars represent the percentage of dye remaining in solution, while red bars correspond to the percentage of dye removed from the solution.

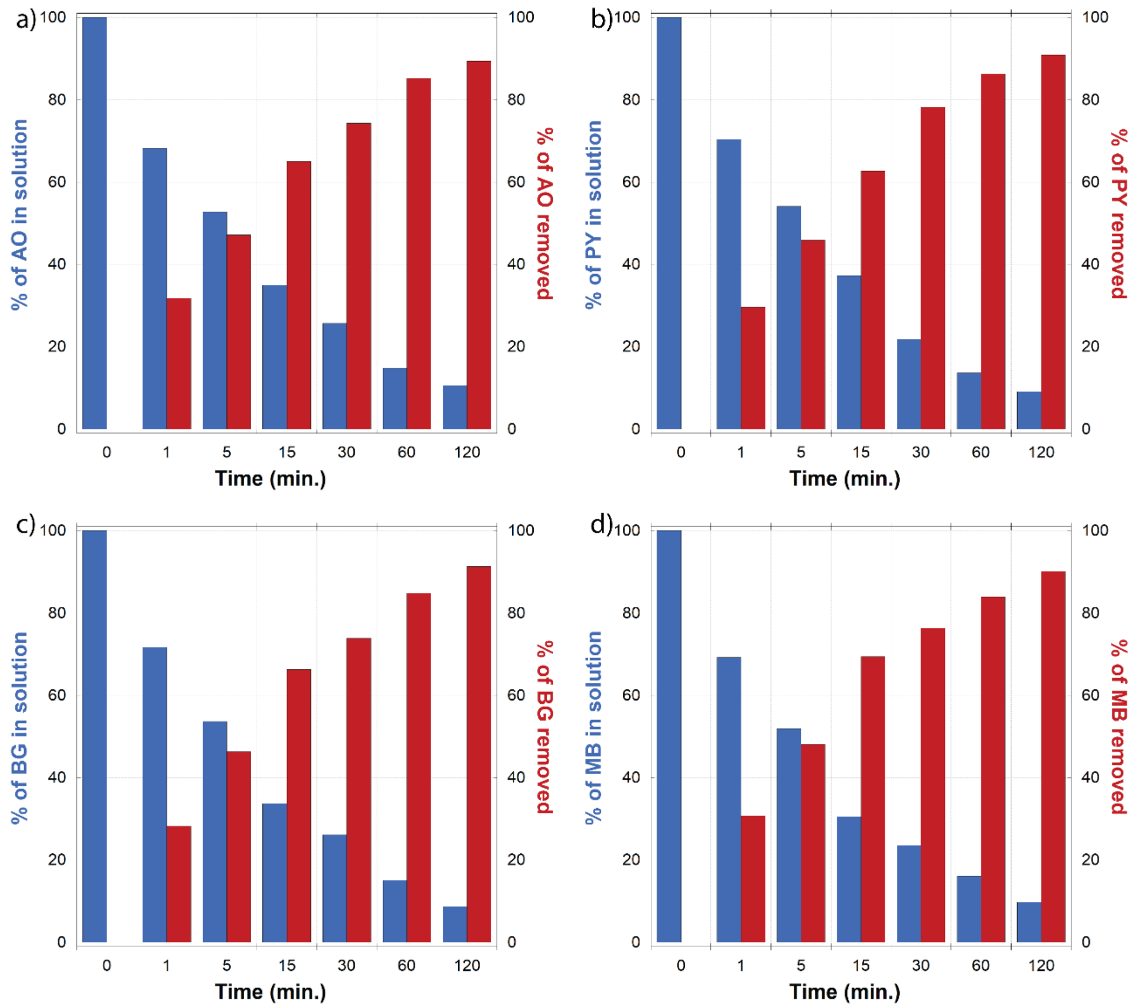

Fig. S21. Time-dependent adsorption of auramine O (AO, a), brilliant green (BG, b), methylene blue (MB, c) and pyronine Y (PY, d) from real water samples collected from the Turia River (Valencia, Spain) using 50 mg of **CAS-1** as the adsorbent during the second adsorption cycle. The initial dye concentration was 10 mg L^{-1} for each dye. Blue bars represent the percentage of dye remaining in solution, while red bars correspond to the percentage of dye removed from the solution.

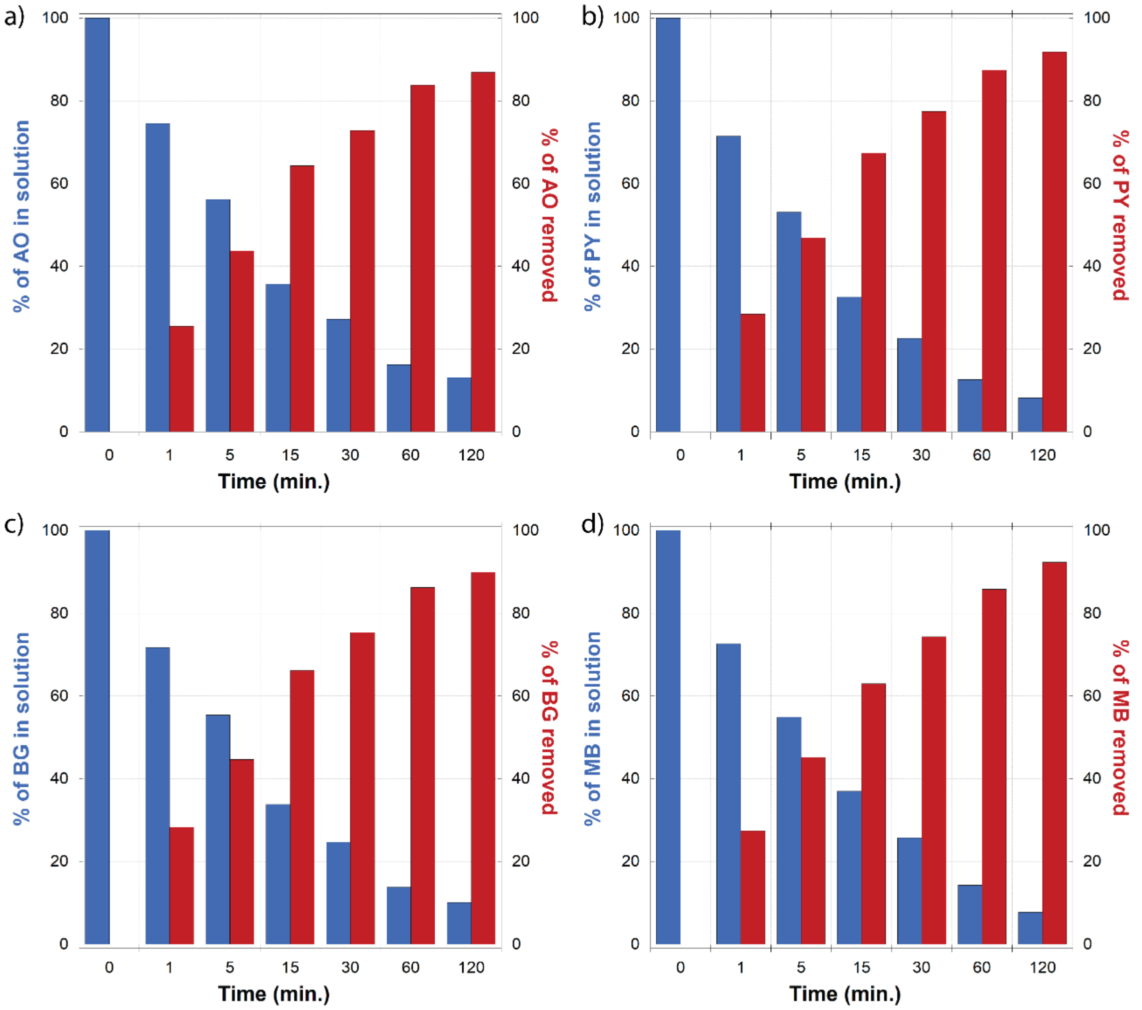

Fig. S22. Time-dependent adsorption of auramine O (AO, a), brilliant green (BG, b), methylene blue (MB, c) and pyronine Y (PY, d) from real water samples collected from the Turia River (Valencia, Spain) using 50 mg of **CAS-1** as the adsorbent during the third adsorption cycle. The initial dye concentration was 10 mg L^{-1} for each dye. Blue bars represent the percentage of dye remaining in solution, while red bars correspond to the percentage of dye removed from the solution.

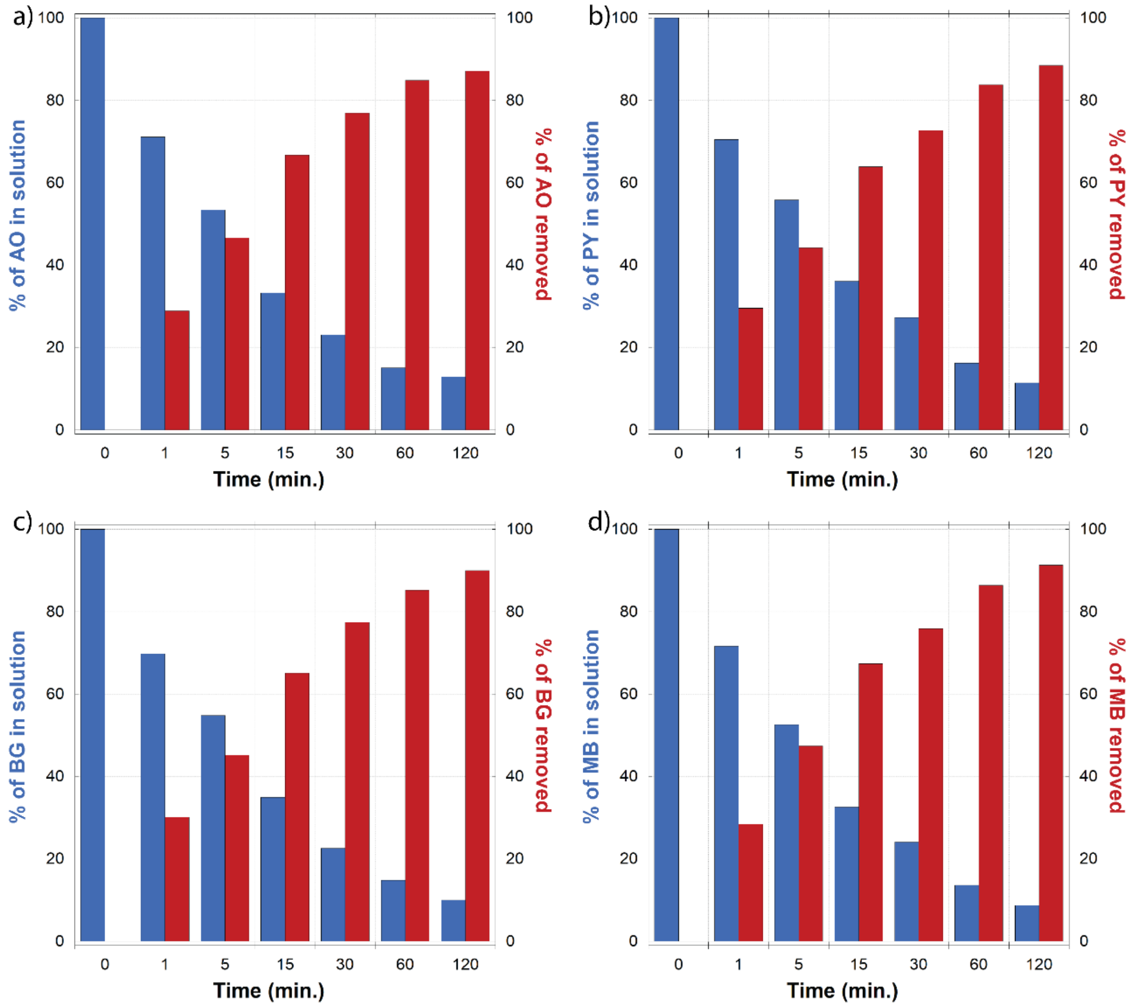

Fig. S23. Time-dependent adsorption of auramine O (AO, a), brilliant green (BG, b), methylene blue (MB, c) and pyronine Y (PY, d) from real water samples collected from the Turia River (Valencia, Spain) using 50 mg of **CAS-1** as the adsorbent during the fourth adsorption cycle. The initial dye concentration was 10 mg L^{-1} for each dye. Blue bars represent the percentage of dye remaining in solution, while red bars correspond to the percentage of dye removed from the solution.

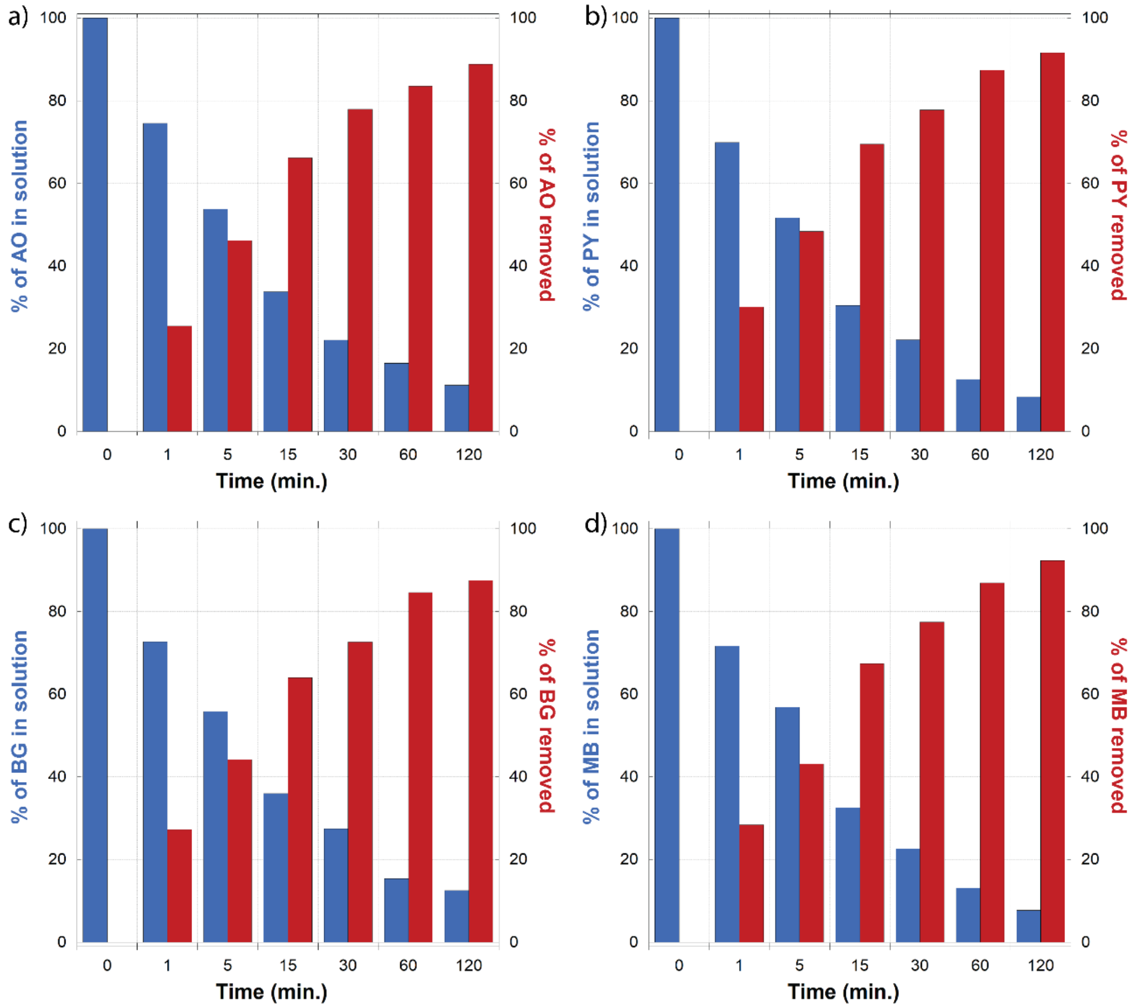

Fig. S24. Time-dependent adsorption of auramine O (AO, a), brilliant green (BG, b), methylene blue (MB, c) and pyronine Y (PY, d) from real water samples collected from the Turia River (Valencia, Spain) using 50 mg of **CAS-1** as the adsorbent during the fifth adsorption cycle. The initial dye concentration was 10 mg L^{-1} for each dye. Blue bars represent the percentage of dye remaining in solution, while red bars correspond to the percentage of dye removed from the solution.

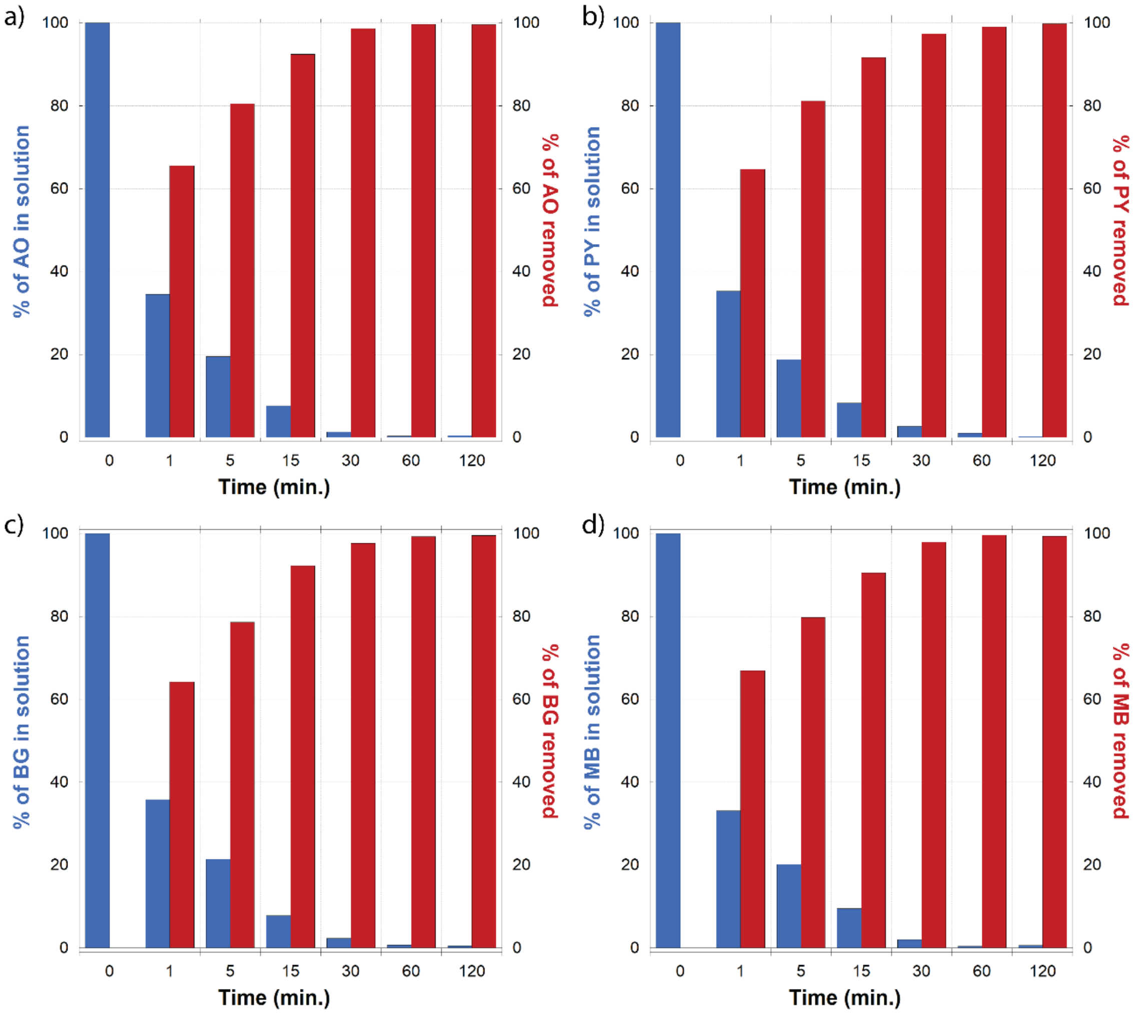

Fig. S25. Time-dependent adsorption of auramine O (AO, a), brilliant green (BG, b), methylene blue (MB, c) and pyronine Y (PY, d) from real water samples collected from the Turia River (Valencia, Spain) using 50 mg of **CAS-2** as the adsorbent during the first adsorption cycle. The initial dye concentration was 10 mg L^{-1} for each dye. Blue bars represent the percentage of dye remaining in solution, while red bars correspond to the percentage of dye removed from the solution.

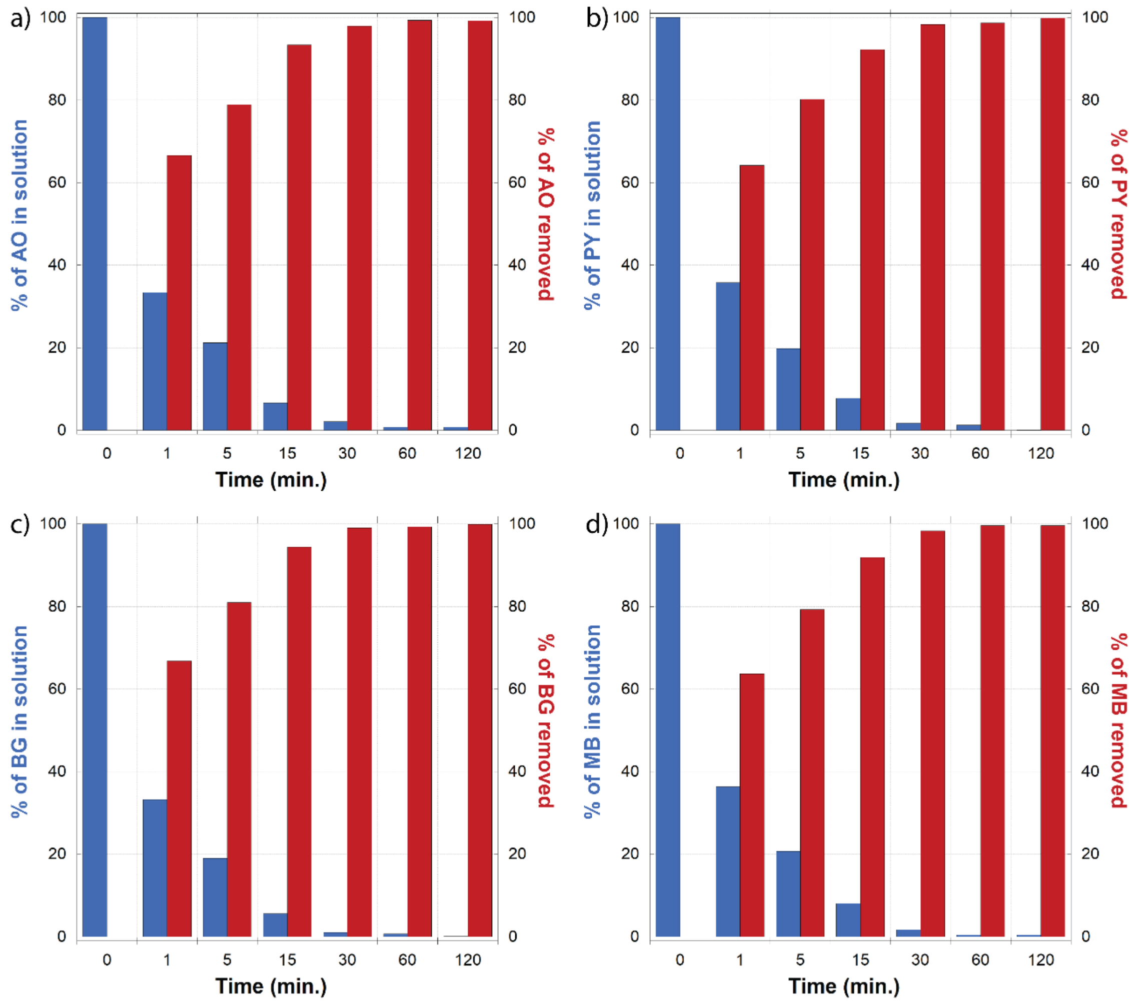

Fig. S26. Time-dependent adsorption of auramine O (AO, a), brilliant green (BG, b), methylene blue (MB, c) and pyronine Y (PY, d) from real water samples collected from the Turia River (Valencia, Spain) using 50 mg of **CAS-2** as the adsorbent during the second adsorption cycle. The initial dye concentration was 10 mg L^{-1} for each dye. Blue bars represent the percentage of dye remaining in solution, while red bars correspond to the percentage of dye removed from the solution.

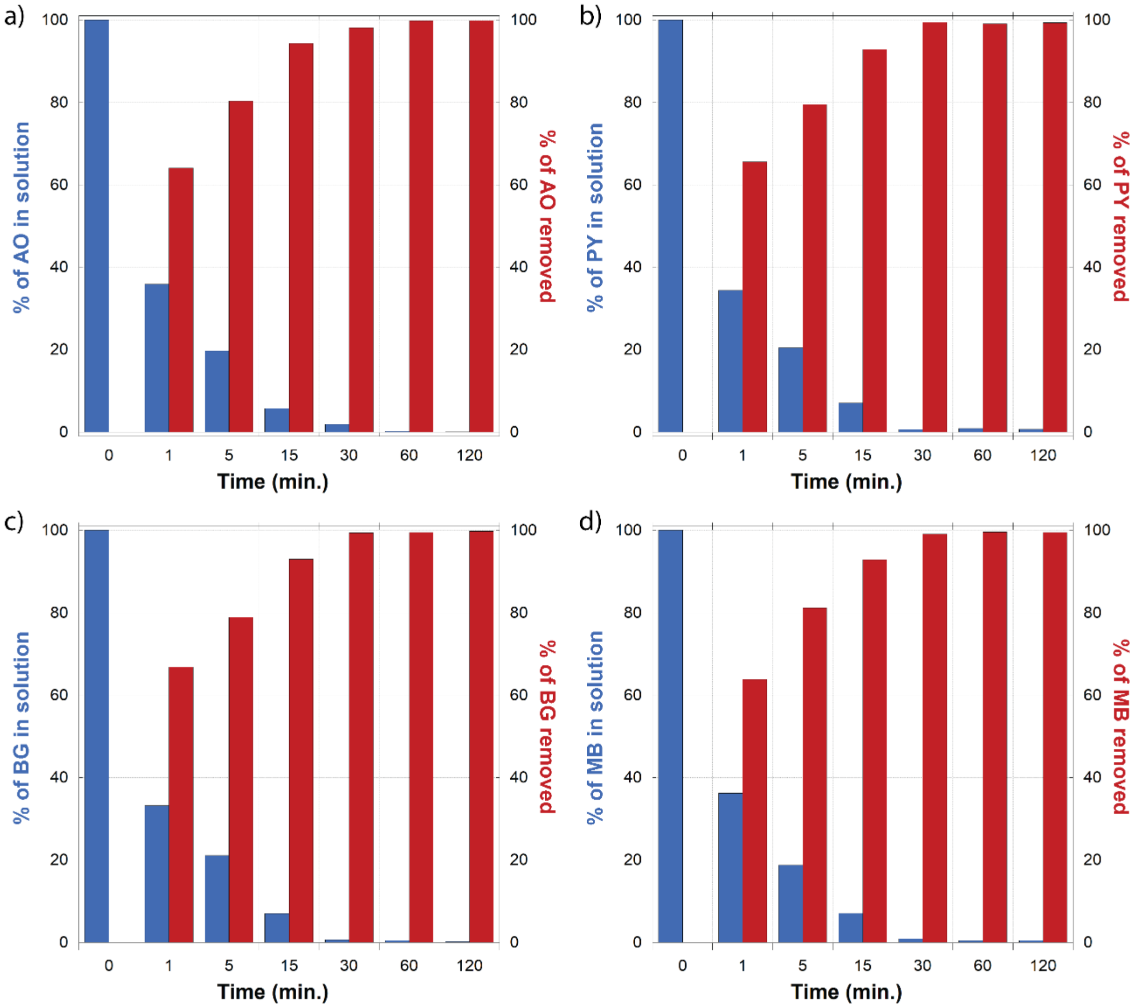

Fig. S27. Time-dependent adsorption of auramine O (AO, a), brilliant green (BG, b), methylene blue (MB, c) and pyronine Y (PY, d) from real water samples collected from the Turia River (Valencia, Spain) using 50 mg of **CAS-2** as the adsorbent during the third adsorption cycle. The initial dye concentration was 10 mg L^{-1} for each dye. Blue bars represent the percentage of dye remaining in solution, while red bars correspond to the percentage of dye removed from the solution.

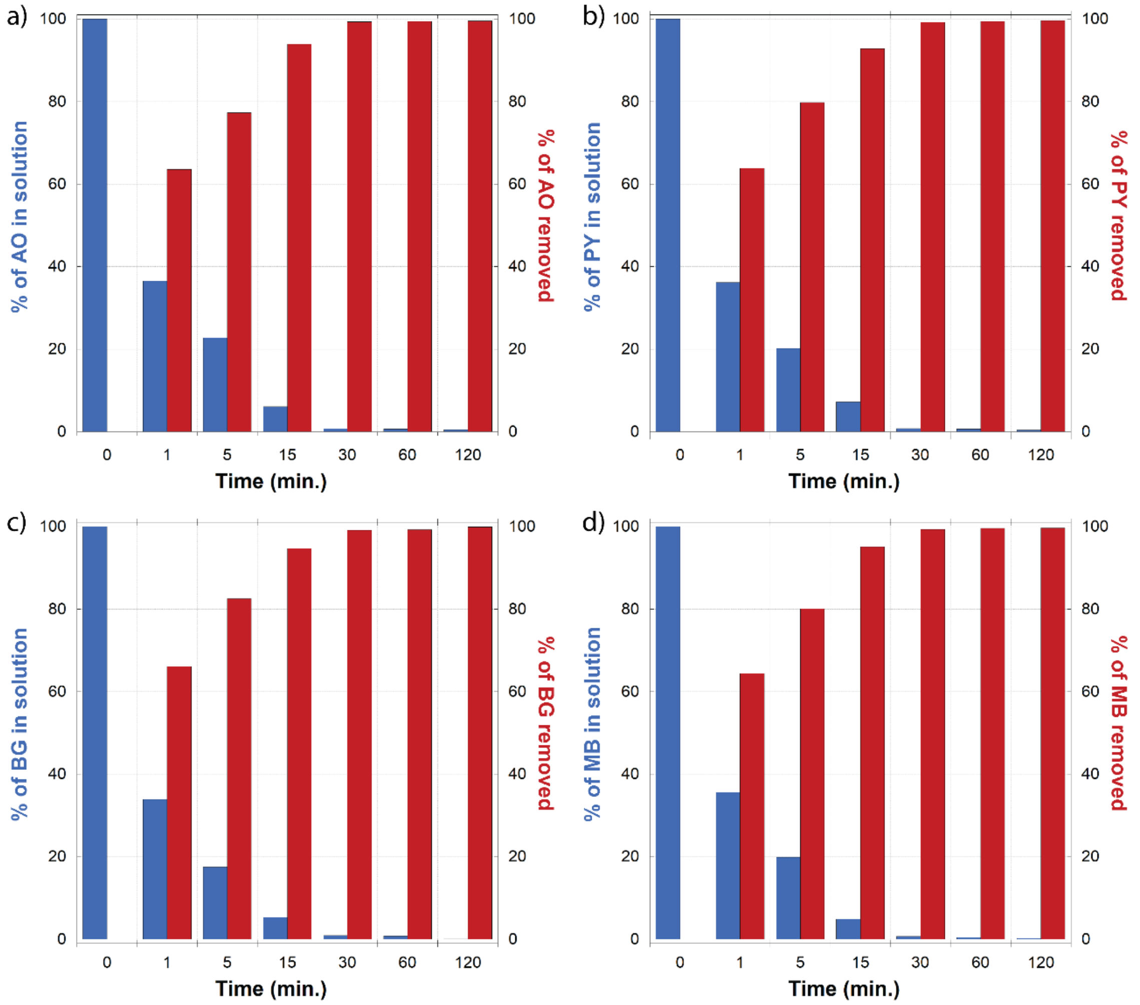

Fig. S28. Time-dependent adsorption of auramine O (AO, a), brilliant green (BG, b), methylene blue (MB, c) and pyronine Y (PY, d) from real water samples collected from the Turia River (Valencia, Spain) using 50 mg of **CAS-2** as the adsorbent during the fourth adsorption cycle. The initial dye concentration was 10 mg L^{-1} for each dye. Blue bars represent the percentage of dye remaining in solution, while red bars correspond to the percentage of dye removed from the solution.

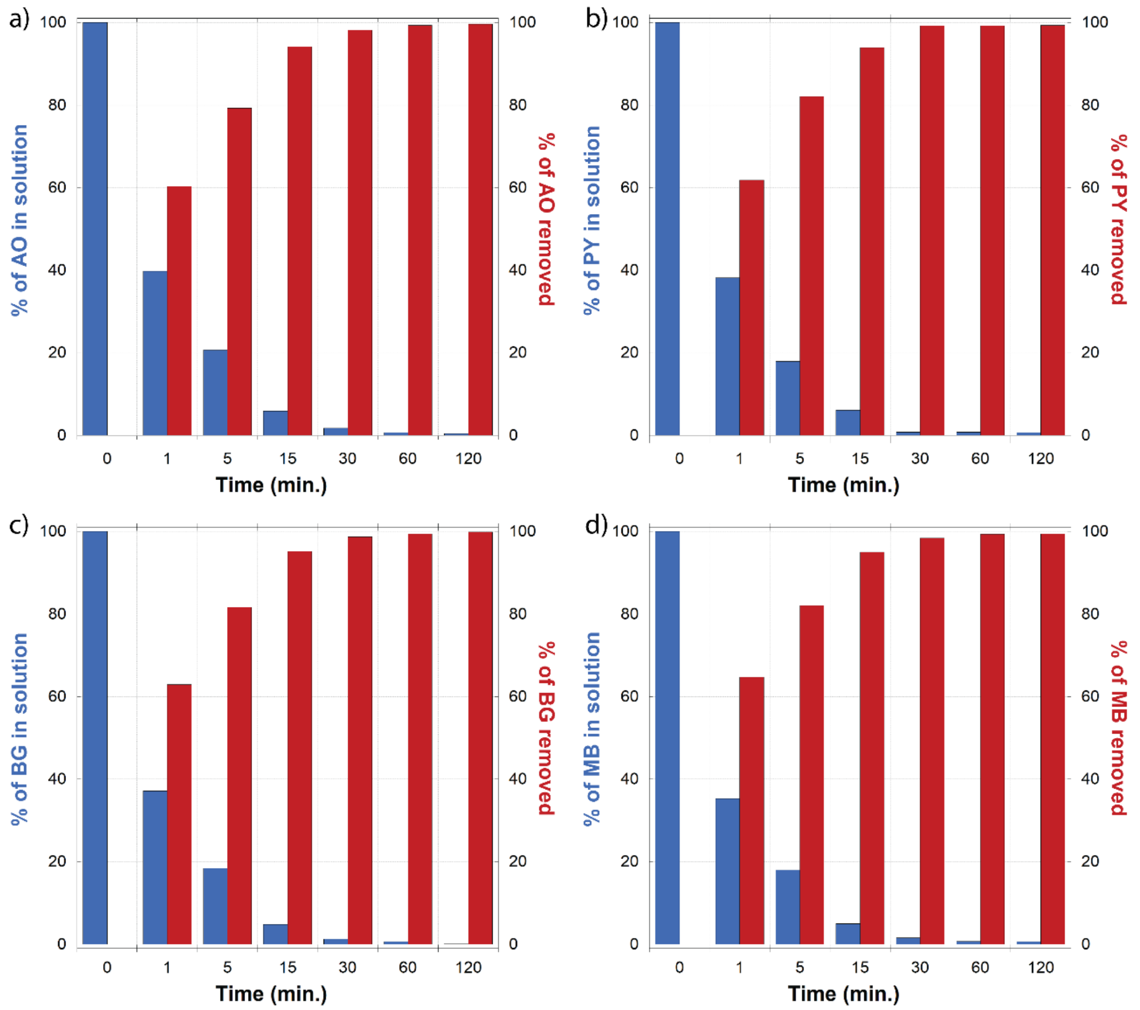

Fig. S29. Time-dependent adsorption of auramine O (AO, a), brilliant green (BG, b), methylene blue (MB, c) and pyronine Y (PY, d) from real water samples collected from the Turia River (Valencia, Spain) using 50 mg of **CAS-2** as the adsorbent during the fifth adsorption cycle. The initial dye concentration was 10 mg L^{-1} for each dye. Blue bars represent the percentage of dye remaining in solution, while red bars correspond to the percentage of dye removed from the solution.

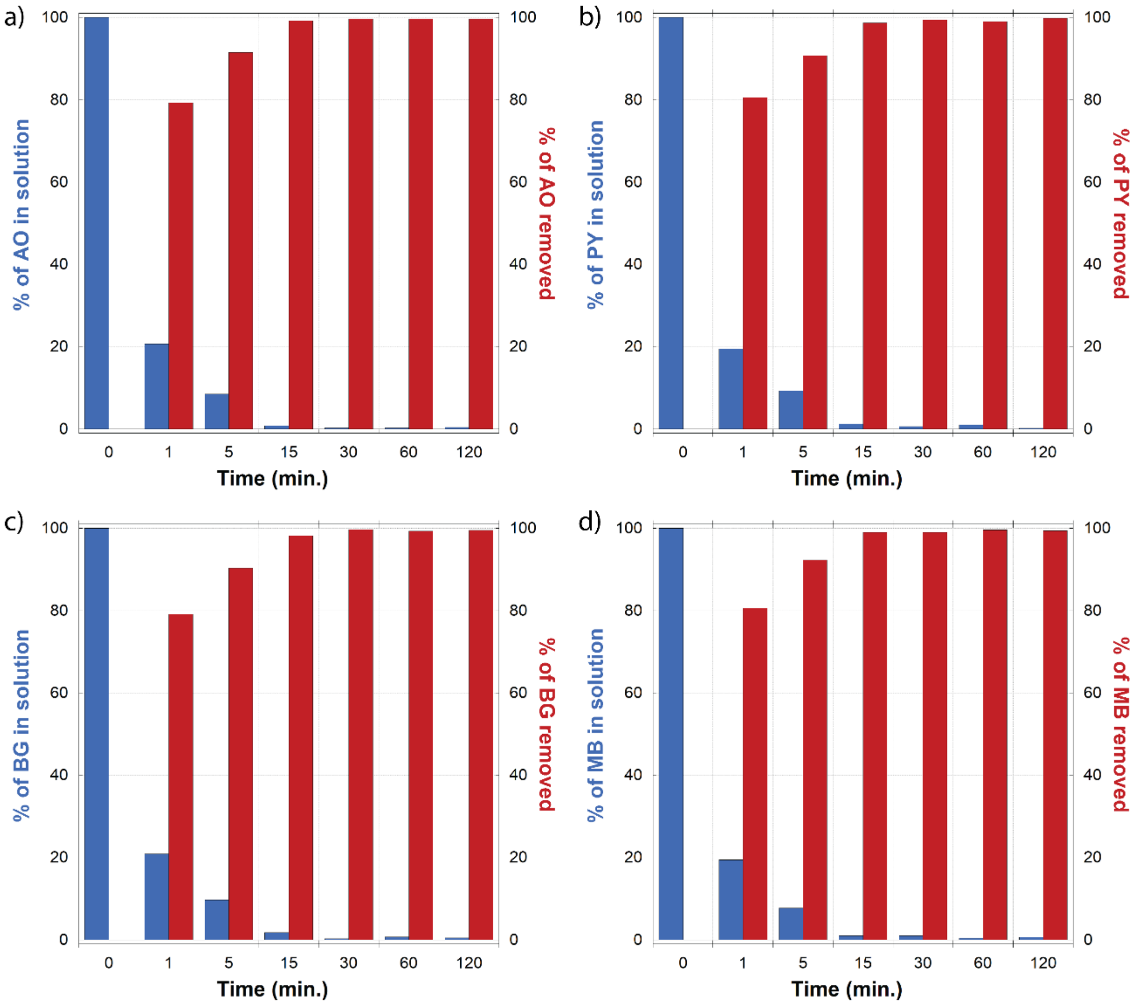

Fig. S30. Time-dependent adsorption of auramine O (AO, a), brilliant green (BG, b), methylene blue (MB, c) and pyronine Y (PY, d) from real water samples collected from the Turia River (Valencia, Spain) using 50 mg of **CAS-3** as the adsorbent during the first adsorption cycle. The initial dye concentration was 10 mg L^{-1} for each dye. Blue bars represent the percentage of dye remaining in solution, while red bars correspond to the percentage of dye removed from the solution.

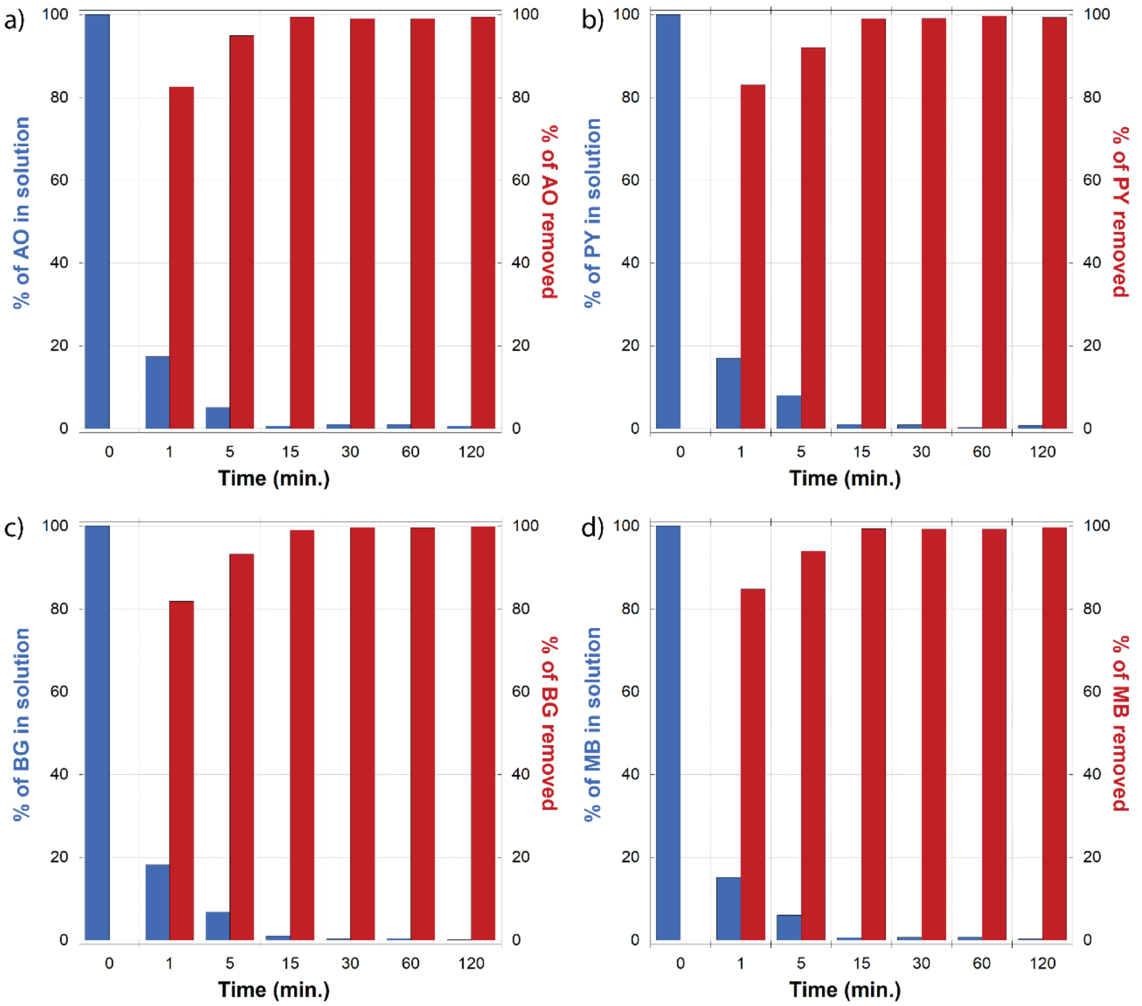

Fig. S31. Time-dependent adsorption of auramine O (AO, a), brilliant green (BG, b), methylene blue (MB, c) and pyronine Y (PY, d) from real water samples collected from the Turia River (Valencia, Spain) using 50 mg of **CAS-3** as the adsorbent during the second adsorption cycle. The initial dye concentration was 10 mg L^{-1} for each dye. Blue bars represent the percentage of dye remaining in solution, while red bars correspond to the percentage of dye removed from the solution.

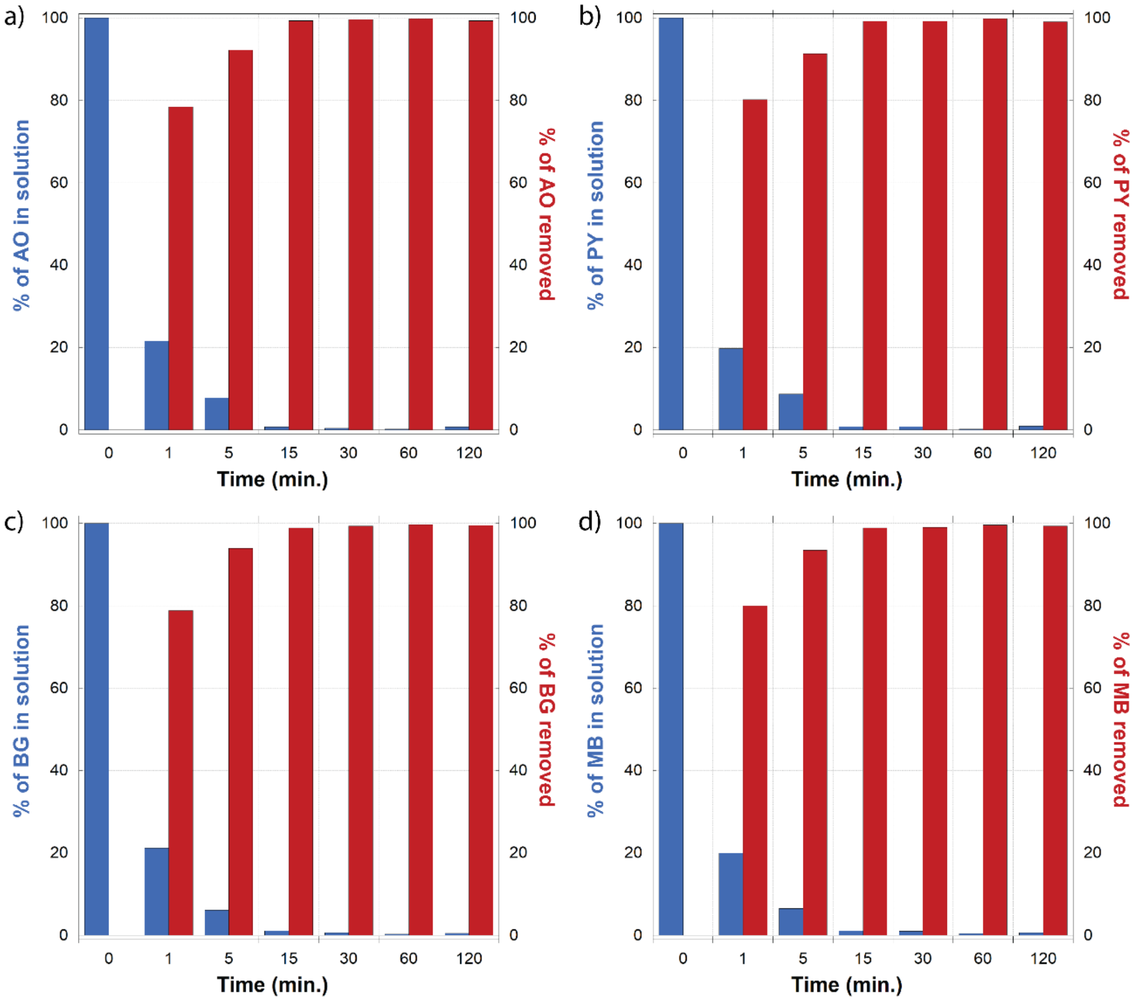

Fig. S32. Time-dependent adsorption of auramine O (AO, a), brilliant green (BG, b), methylene blue (MB, c) and pyronine Y (PY, d) from real water samples collected from the Turia River (Valencia, Spain) using 50 mg of **CAS-3** as the adsorbent during the third adsorption cycle. The initial dye concentration was 10 mg L^{-1} for each dye. Blue bars represent the percentage of dye remaining in solution, while red bars correspond to the percentage of dye removed from the solution.

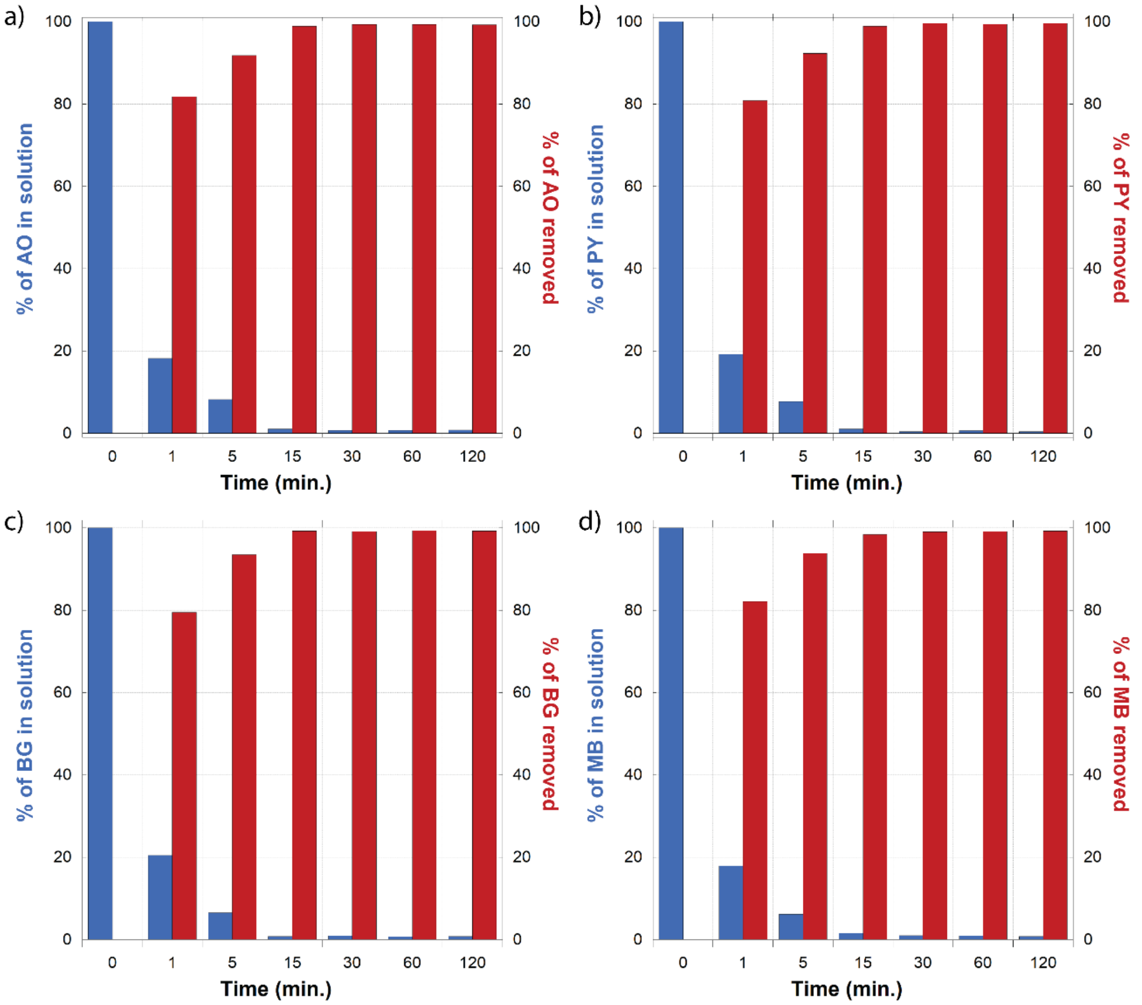

Fig. S33. Time-dependent adsorption of auramine O (AO, a), brilliant green (BG, b), methylene blue (MB, c) and pyronine Y (PY, d) from real water samples collected from the Turia River (Valencia, Spain) using 50 mg of **CAS-3** as the adsorbent during the fourth adsorption cycle. The initial dye concentration was 10 mg L^{-1} for each dye. Blue bars represent the percentage of dye remaining in solution, while red bars correspond to the percentage of dye removed from the solution.

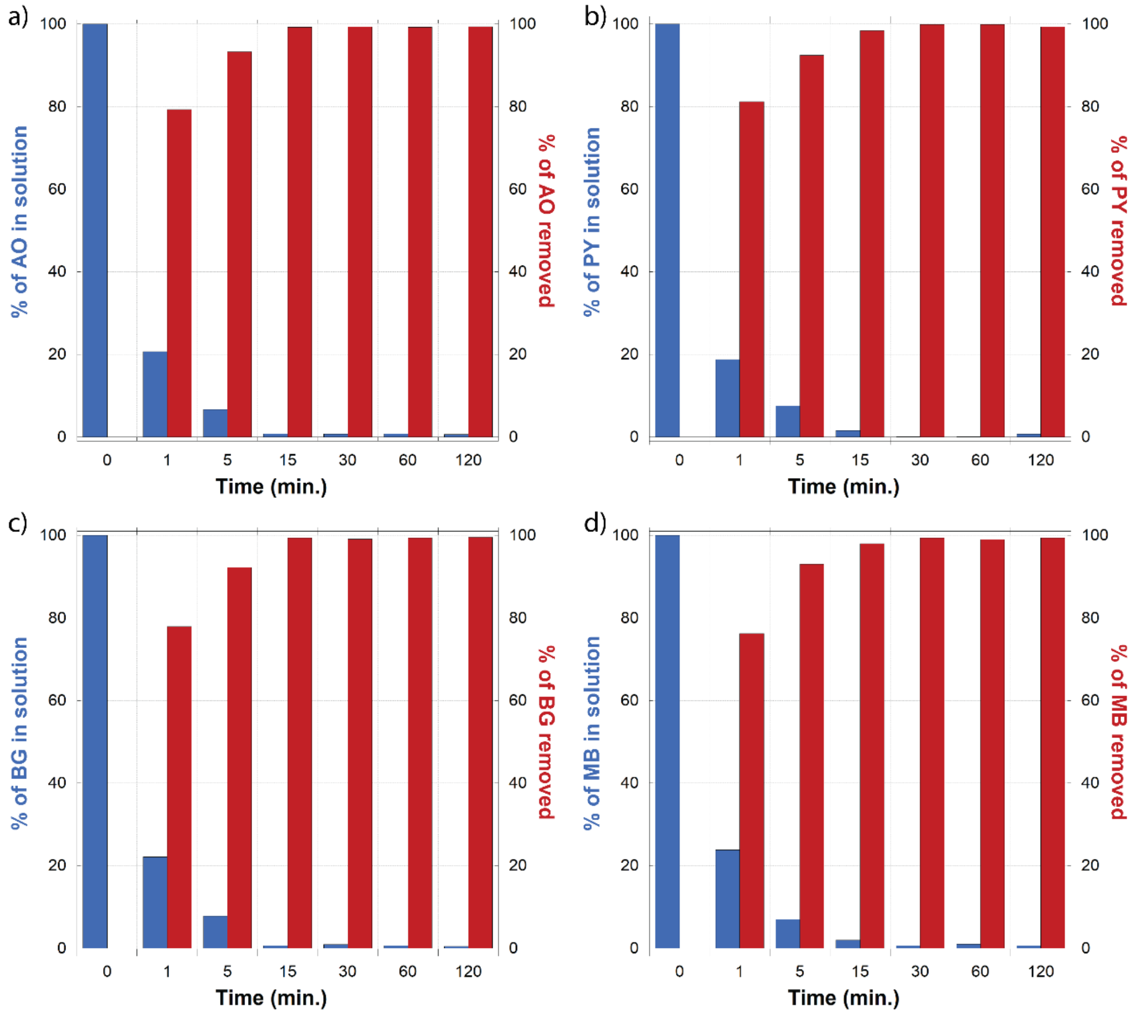

Fig. S34. Time-dependent adsorption of auramine O (AO, a), brilliant green (BG, b), methylene blue (MB, c) and pyronine Y (PY, d) from real water samples collected from the Turia River (Valencia, Spain) using 50 mg of **CAS-3** as the adsorbent during the fifth adsorption cycle. The initial dye concentration was 10 mg L^{-1} for each dye. Blue bars represent the percentage of dye remaining in solution, while red bars correspond to the percentage of dye removed from the solution.

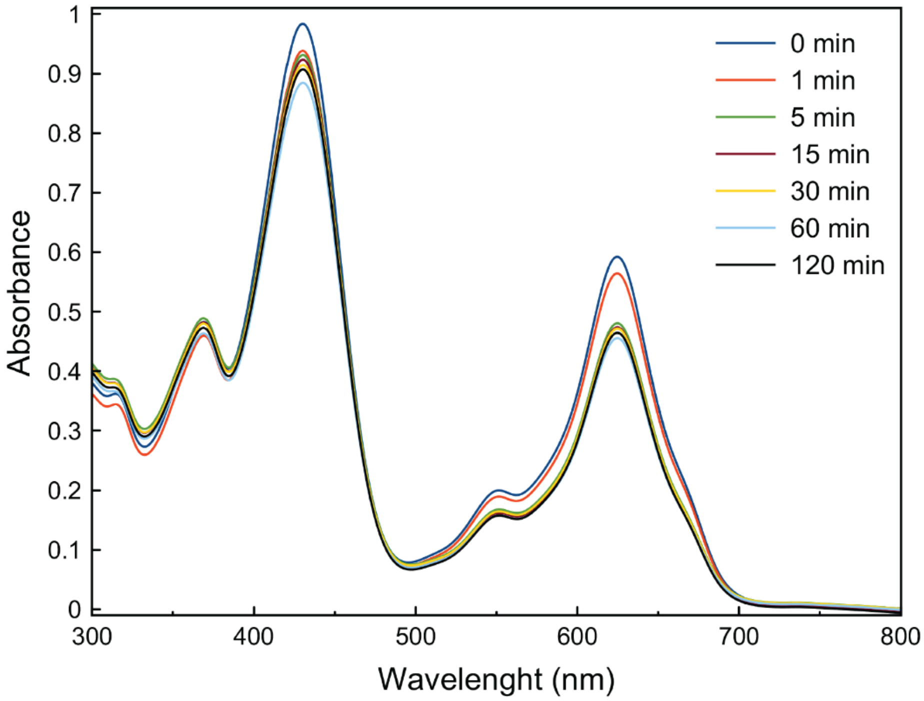

Fig. S35. Evolution with time of the UV-Vis absorption spectra of a multidye solution containing 10 ppm solutions of Auramine O, Brilliant green, Methylene blue and Pyronin Y in real water samples from Turia river in the presence of 50 mg of pristine calcium alginate spheres.

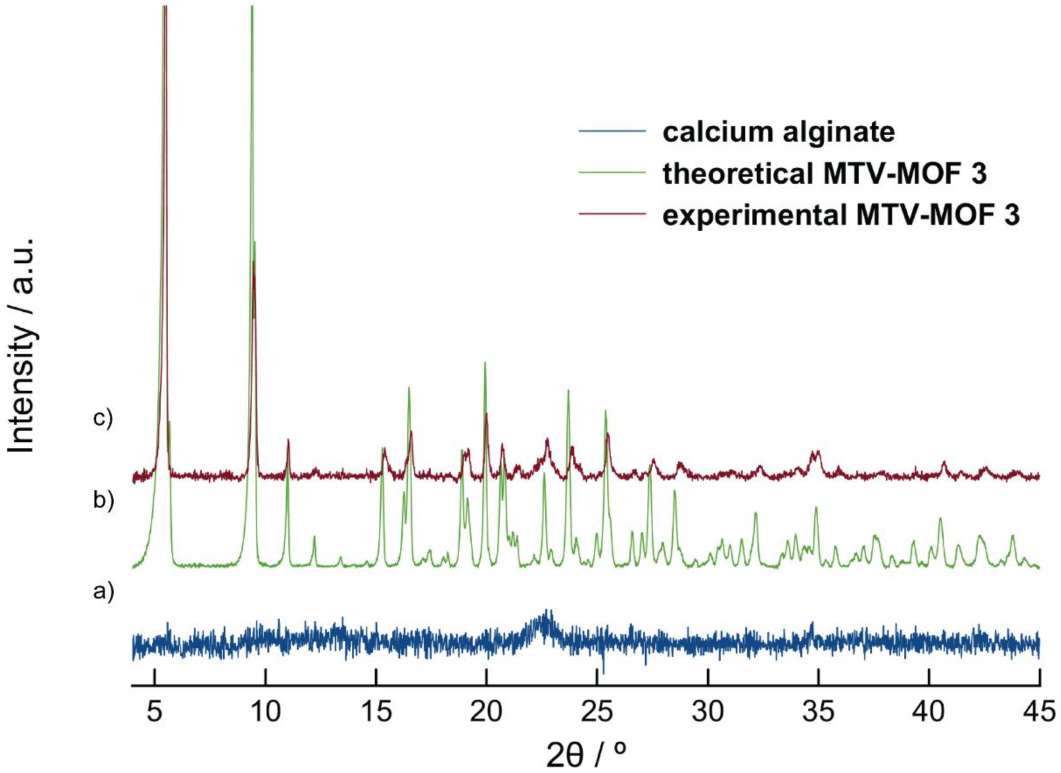

Fig. S36. (a) Experimental PXRD pattern profile of pristine calcium alginate spheres**.** Theoretical (b) and experimental after 5 capture cycles (c) PXRD pattern profiles of **CAS-3** in the 2θ range 3.0–45.0.

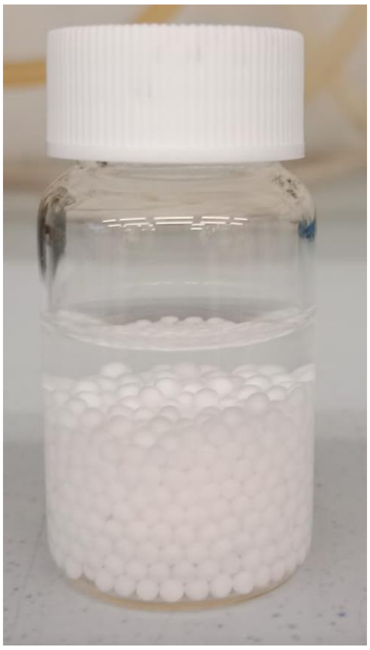

Fig. S37. Photograph of calcium alginate spheres containing MTV-MOF **3** (**CAS-3**) after prolonged storage in water (3 months), illustrating their macroscopic integrity.

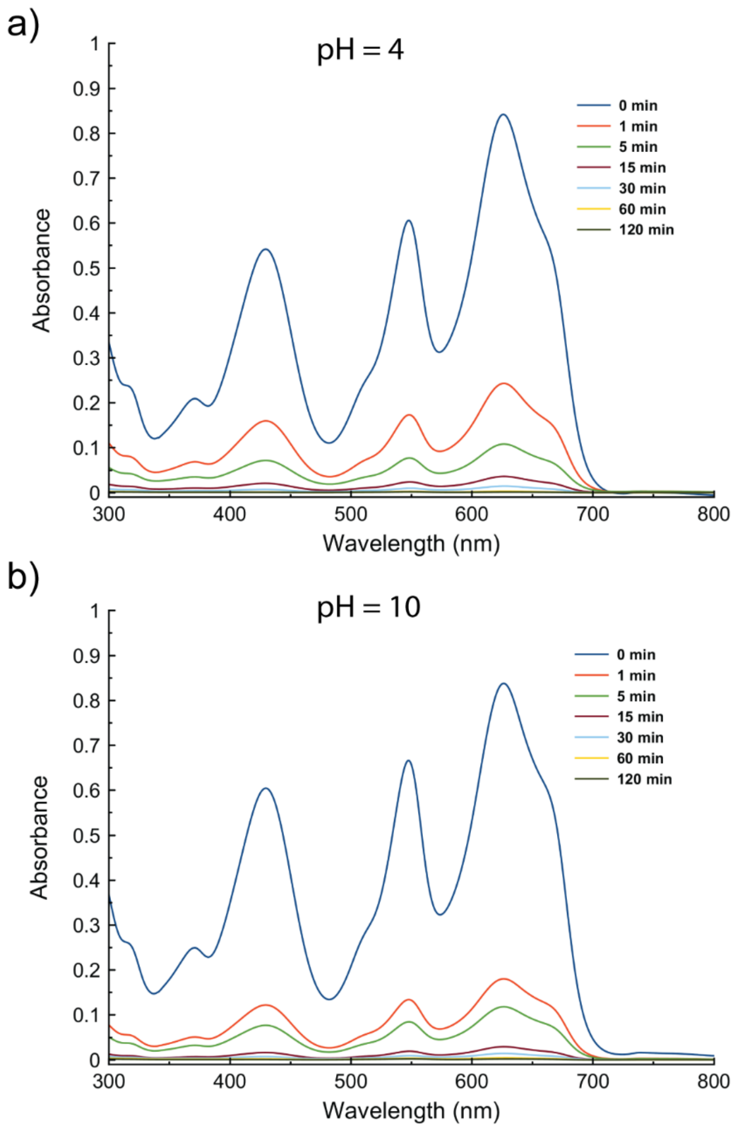

Fig. S38. Evolution with time of the UV-Vis absorption spectra of a multidye solution containing 10 ppm solutions of Auramine O, Brilliant green, Methylene blue and Pyronin Y in real water samples from Turia river, at pH = 4 (a) and pH = 10, in the presence of 50 mg of **CAS-3**. pH values were adjusted with HCl and NaOH, respectively.

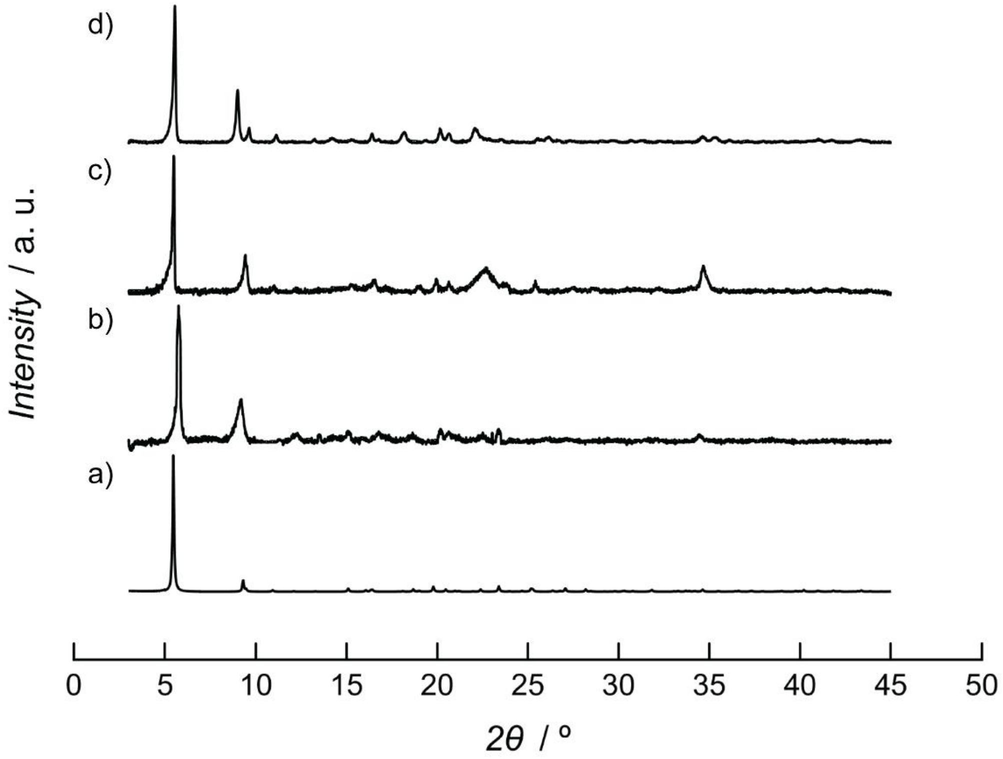

Fig. S39. Theoretical PXRD pattern of MTV-MOF **3** (a) and experimental PXRD pattern profile of **CAS-3** after capture experiments at pH = 7.4 (b), pH = 4 (c) and pH = 10 (d), in the 2θ range 3.0–45.0.

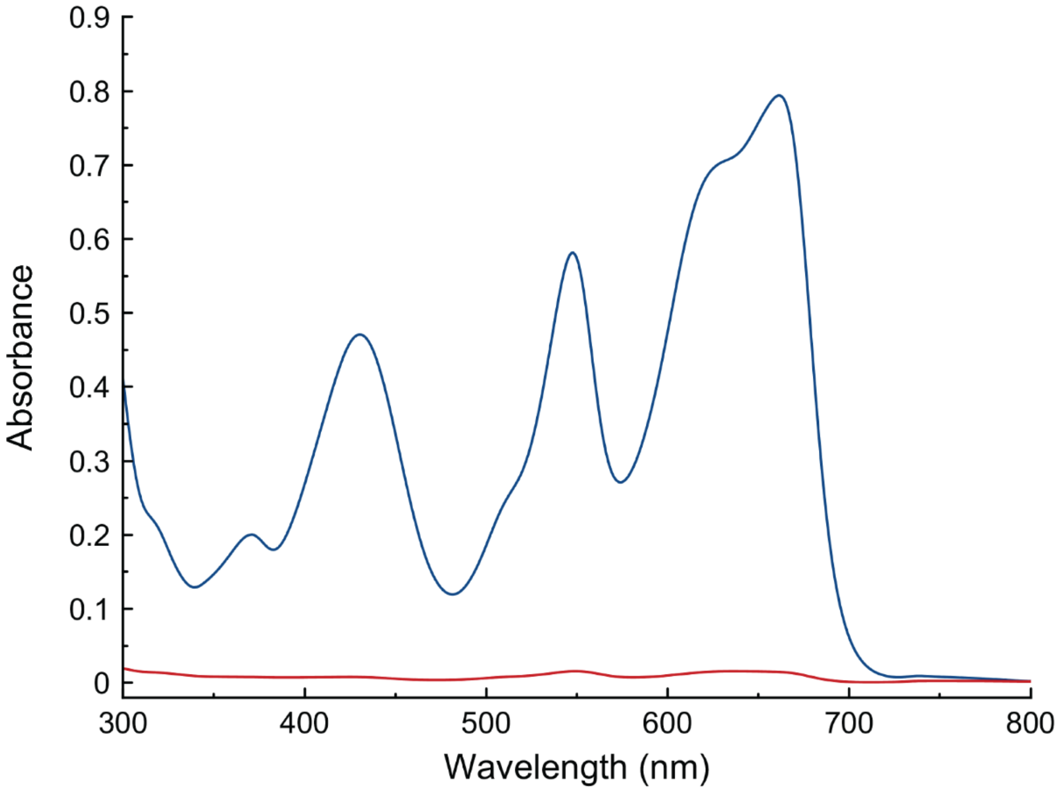

Fig. S40. UV–Vis absorption spectra of a multidye solution containing 10 mg L^{-1} each of Auramine O, Brilliant Green, Methylene Blue, and Pyronin Y in real water samples from the Turia River, in the presence of 25 mg of **CAS-3**, before (blue line) and after (red line) being percolated (continuous-flow conditions) through a solid-phase extraction (SPE) setup.

Bibliography

1 P. Escamilla, L. Bartella, S. Sanz-Navarro, R. M. Percoco, L. Di Donna, M. Prejanò, T. Marino, J. Ferrando-Soria, D. Armentano, A. Leyva-Pérez and E. Pardo, *Chem. – A Eur. J.*, , , DOI:10.1002/chem.202301325.

2 P. Escamilla, M. Monteleone, R. M. Percoco, T. F. Mastropietro, M. Longo, E. Esposito, A. Fuoco, J. C. Jansen, R. Elliani, A. Tagarelli, J. Ferrando-Soria, V. Amendola, E. Pardo and D. Armentano, *ACS Appl. Mater. Interfaces*, 2024, **16**, 51182–51194.

3 M. Baratta, T. F. Mastropietro, P. Escamilla, V. Algieri, F. Xu, F. P. Nicoletta, J. Ferrando-Soria, E. Pardo, G. De Filpo and D. Armentano, *Inorg. Chem.*, 2024, **63**, 18992–19001.

4 C. F. Macrae, I. J. Bruno, J. A. Chisholm, P. R. Edgington, P. McCabe, E. Pidcock, L. Rodriguez-Monge, R. Taylor, J. van de Streek and P. A. Wood, *J. Appl. Crystallogr.*, 2008, **41**, 466–470.
